# Automated content analysis across six languages

**DOI:** 10.1371/journal.pone.0224425

**Published:** 2019-11-20

**Authors:** Leah Cathryn Windsor, James Grayson Cupit, Alistair James Windsor

**Affiliations:** 1 Institute for Intelligent Systems, The University of Memphis, Memphis, Tennessee, United States of America; 2 Institute for Intelligent Systems, The University of Memphis, Memphis, Tennessee, United States of America; 3 Department of Mathematical Sciences, The University of Memphis, Memphis, Tennessee, United States of America; University of California Santa Barbara, UNITED STATES

## Abstract

Corpus selection bias in international relations research presents an epistemological problem: How do we know what we know? Most social science research in the field of text analytics relies on English language corpora, biasing our ability to understand international phenomena. To address the issue of corpus selection bias, we introduce results that suggest that machine translation may be used to address non-English sources. We use human translation and machine translation (Google Translate) on a collection of aligned sentences from United Nations documents extracted from the Multi-UN corpus, analyzed with a “bag of words” analysis tool, Linguistic Inquiry Word Count (LIWC). Overall, the LIWC indices proved relatively stable across machine and human translated sentences. We find that while there are statistically significant differences between the original and translated documents, the effect sizes are relatively small, especially when looking at psychological processes.

## Introduction

The motivation for this paper is to address the lack of linguistic diversity among text-as-data scholarship by quantifying the effects of machine translation on linguistic measures across a variety of languages. The primary goal is not to determine whether there are statistically significant differences, as for most indices there are, but rather to determine the effect size of the machine translation. This will enable future researchers to determine whether an observed effect might reasonably be attributed to the effects of translation or whether an alternative explanation, such as corpus irregularities, is more plausible. We employ the MultiUN corpus to test the stability of LIWC (Linguistic Inquiry Word Count) measures across machine and human translation because it was developed specifically to facilitate the improvement of machine translation techniques.

LIWC is a useful tool for identifying linguistic patterns, threatening language and deception [1,2], gendered language [3], social meaning and personality [4], and hierarchy and status in opaque political groups [5]. At present, there is a corpus diversity problem in computational discourse analysis for social science research questions. Most of the readily available corpora exist for English-language and Western sources, as document preservation, archiving, and formatting tend to face more technological hurdles in non-Western countries. In addition, most computational linguistics programs only work on English-language corpora. Providing evidence for multilingual translational stability across LIWC measures can help broaden the diversity of document sources. In turn, this may facilitate more representative analysis across languages and cultures, increasing our knowledge about language and politics beyond Western-style institutions.

Because the MultiUN corpus provides expert translations into, or between, the UN’s official languages, it provides an unparalleled source for comparing the LIWC indices of machine translated sentences against the LIWC indices of human translated documents for five non-English languages simultaneously. We find that while there are statistically significant differences between machine and human translated documents across LIWC indices, the effect sizes are very small. This means that scholars can reliably use Google Translate alongside off-the-shelf, bag-of-words analysis programs for political science research.

The structure of this paper proceeds as follows. We first provide an orientation the problems researchers face in dealing with multilingual corpora and discuss some of the relevant literature on this topic. We then describe the methodology of our analysis of the effects of machine translation on LIWC indices. Following this, we present our results, including the substantive differences between human and computer-translated documents. We conclude with a discussion of how using machine translation can impact the scope of social science inquiry.

## Background

Much of quantitative text analysis for political research is done using English-language sources, introducing corpus selection bias at the level of the data generating process that may influence the outcome of results [6]. Additionally, most computational linguistics programs only work with English-language data. This further limits the universe of cases from which researchers can generate corpora, of particular concern to scholars of international relations and comparative politics [7]. To address the issue of corpus bias, we introduce results that show that for many LIWC indices machine translation and human translation yield very similar results. We simultaneously address French, German, Russian, Mandarin, and Arabic [8]. Linguistic analysis programs such as LIWC (Linguistic Inquiry and Word Count) have been used to explore phenomena such as deception detection [2], radicalization [9], diplomacy [10,11], and populism and presidential popularity [12]. By expanding options for corpus selection, we can broaden the analysis of political texts to observe and analyze phenomena from the non-English speaking world [13,14].

Broadly speaking, the field of “text as data" has become well-established in political science scholarship, particularly in the field of American politics, using English-language corpora [15–23]. Though computational text analysis has lagged behind in the field of international relations, scholars are increasingly using text-as-data approaches to understand issues like censorship in social media [13,24], crises in authoritarian regimes [25], foreign policy in state media [14], and leaders’ resolve [26,27]. While some computational methods, such as Latent Dirichlet Allocation [28], can process text in many languages [29], other programs like Coh-Metrix [30], Seance [31], Lexicoder [32], and LIWC (Linguistic Inquiry and Word Count) [33] are limited in their ability to analyze non-English texts.

In the case of LIWC, this limitation is due to the reliance on curated word lists to compute its indices. In the case of Coh-Metrix this limitation arises from the use of word lists for some indices such as lists of connectives and age-of-acquisition lists, from the reliance on parsing (e.g., the current German parser does not distinguish adverbs and adjectives), and from the dependence of some indices on English grammatical constructs. For example, left-embeddedness does not make sense as an index for S-O-V languages [34].

### Problems and solutions

Given the language limitations of computational linguistics programs, one potential solution is to employ expert human translators to translate the documents from the original language to the target language, usually English. Expert human translation, however, is both expensive and time-consuming. Automated translation programs, on the other hand, are efficient and cost-effective [35,36], and other research has shown encouraging results using machine learning for automated sentiment analysis for multilingual corpora [37], including spam filtering [38,39]. Given limited time and money, researchers have had to choose between greater accuracy, greater efficiency, and greater expense. To help resolve this research quandary, this paper explores whether LIWC measures under machine translation are similar to those of huma translation [8].

An area of concern in machine translation is whether the computer program is able to approximate the skill and accuracy of human translation [35,40,41]. A range of software options are available for automated translation, but using Google Translate is advantageous because it is low-cost, user-friendly, and relatively reliable for many languages. Recent research in has established that Google Translate is a reliable process for bag-of-words approaches to text analysis, such as topic modeling [42]. Our approach of LIWC measures offers a corollary analysis alongside the work of de Vries et al., 2018. At the time that our documents were translated, Google Translate used a Phrase-Based Machine Translation algorithm for all but Standard Chinese for which it used the new Google Neural Machine Translation algorithm.

Checking the stability of a linguistic measure under machine translation is difficult. One obviously wants to take the same sentence in two different languages, but what does this mean? There are four natural approaches [40,42,43]: first, take a sentence in one language and translate it to another language using both machine and human translators; second, take a sentence in one language, translate it to another language using a human translator, and then translate the translated sentence back to the initial language using the machine translator [35]; third, take a sentence in one language, translate it to another language using the machine translator, and then translate the translated sentence back to the initial language using a human translator; and fourth, take a sentence in one language, translate it to another language using the machine translator, and then translate the translated sentence back to the initial language using the machine translator.

In our workflow, we eliminate the last two possibilities. Of the two involving translating and then retranslating the translated sentence, we prefer to apply the human translator first. Human translators remain the gold standard, so we prefer to input the “better'' human translation to the machine translator, rather than have the human struggle with the lower fidelity machine translation. The fourth possibility seems to test the reversibility of the translation algorithm rather than its fidelity. One can imagine an algorithm which is highly reversible, but which nonetheless provides translations of low fidelity. This leaves us with the first two possibilities. The nature of our corpus means that we in fact employ both strategies (and a fifth strategy where a sentence in one language is human translated into a second and third language and then machines translated from the second into the third) and cannot say which we are employing for any given sentence.

Skeptics might identify an issue using the MultiUN corpus, namely that it likely was included in the document set to train Google Translate algorithms. It is difficult to address this concern given that Google does not make the training data for their translation algorithms public. It is probable that our sample from the MultiUN corpus was indeed included in the training set as it is one of the “gold standard parallel corpora.” However, because it would likely represent such a small proportion of the total available United Nations documents used to generate translation algorithms, the effect would likely be insignificant.

The accuracy of machine translations is often scored using the bilingual evaluation understudy (BLEU) metric, used to evaluate the quality of text that has been machine-translated from one natural language to another [44]. The BLEU metric has been criticized since there are frequently different valid translations and BLEU relies on exact word matching. However, in using LIWC to analyze the translation we will score a match if one word in the angry dictionary is replaced by another word in the angry dictionary. Thus, our LIWC metrics are more forgiving while still capturing dimensions of interest to social scientists.

There is some debate about the use of off-the-shelf, dictionary-based, bag-of-words sentiment analysis programs such as LIWC [45]. Young and Soroka demonstrate that LIWC has the highest correlation (.753) with the Lexicoder Sentiment Dictionary used in social science research, compared with eight other automated sentiment analysis programs [32]. We suggest that the strength of programs like LIWC is the consistency of results that allow theoretically aligned, apples-to-apples comparisons across analyses, given the same stable, transparent dictionaries [4]. Using LIWC alongside other approaches such as topic modeling, syntactic, and semantic analyses can provide a well-rounded picture of political language phenomena.

## Methodology

Given that LIWC takes a "bag of words" approach to computational linguistics analysis, meaning it is not dependent on word order or syntax, can automated translation approximate the reliability associated with human translation? Using the MultiUN data set, we address this question and find that while there are statistically significant differences between the LIWC scoring of machine and human translations, the effect sizes are quite small. In other words, we can say with confidence that the error associated with LIWC results is sufficiently tiny that any substantial differences in LIWC values should be attributed to the corpus, not the translation.

### Corpus description

**Fig 1:**
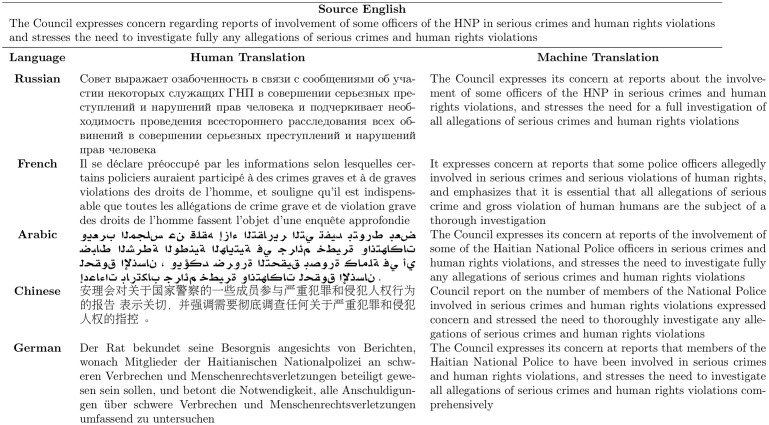
Example of parallel machine translation.

The MultiUN corpus aligns parallel translations across meaning units. Fig 1 provides a sample sentence from the MultiUN dataset, showing the English sentence, its human translation into other languages, and the machine translation back to English that our data provides. The MultiUN documents are in Chinese, English, Russian, Arabic, German, and French (with occasional Spanish documents which we have ignored for this analysis, due to their scarcity in the source corpus).

Each parallel sentence in the MultiUN corpus is represented in the corpus map as a link, and each document as a list of links called a link group. A link defines the position of sentences in their language’s respective corpora as well as a certainty score. This certainty score is an output of the hunalign algorithm used to automatically align the individual sentences [46]. Hunalign is an algorithm specifically developed for cross-language text alignment and was used to produce the Multi-UN corpus in its present form.

### Document preparation

To facilitate navigating and searching through MultiUN’s large XML files, the first step was to insert the link groups from every corpus map into a document-oriented database. To that end, we constructed an event-based XML parser to read through the corpus map files and populate a MongoDB database with link groups and their constituent links.

We then constructed a list of every unique document that had a representation in all of the following languages: English, French, German, Chinese, Russian, and Arabic. For each unique document in the resulting list, we filtered the link group based on two criteria. First, we excluded any sentence whose English representation was fewer than 140 characters. This thresholding was performed to avoid sampling headers, footers, and other such artifacts present in UN documents that do not express a unit of speech. Secondly, we excluded any sentence whose certainty score > 0.5. Initially, we tried excluding any sentence whose certainty did not fall between the 60th and 80th percentile of the entire MultiUN corpus; however, it was observed that sentences with certainties higher than 0.5 skewed heavily towards formulaic, procedural statements (e.g., *Recalling its previous resolutions concerning the situation in Somalia*, *in particular resolution 733 (1992) of 23 January 1992* …), due to the hunalign algorithm finding increased certainty in numerical and ordinal language [46]. Thus, our certainty constraint, despite seeming at first counterintuitive, was actually necessary to focus on the more content rich sentences.

**Fig 2:**
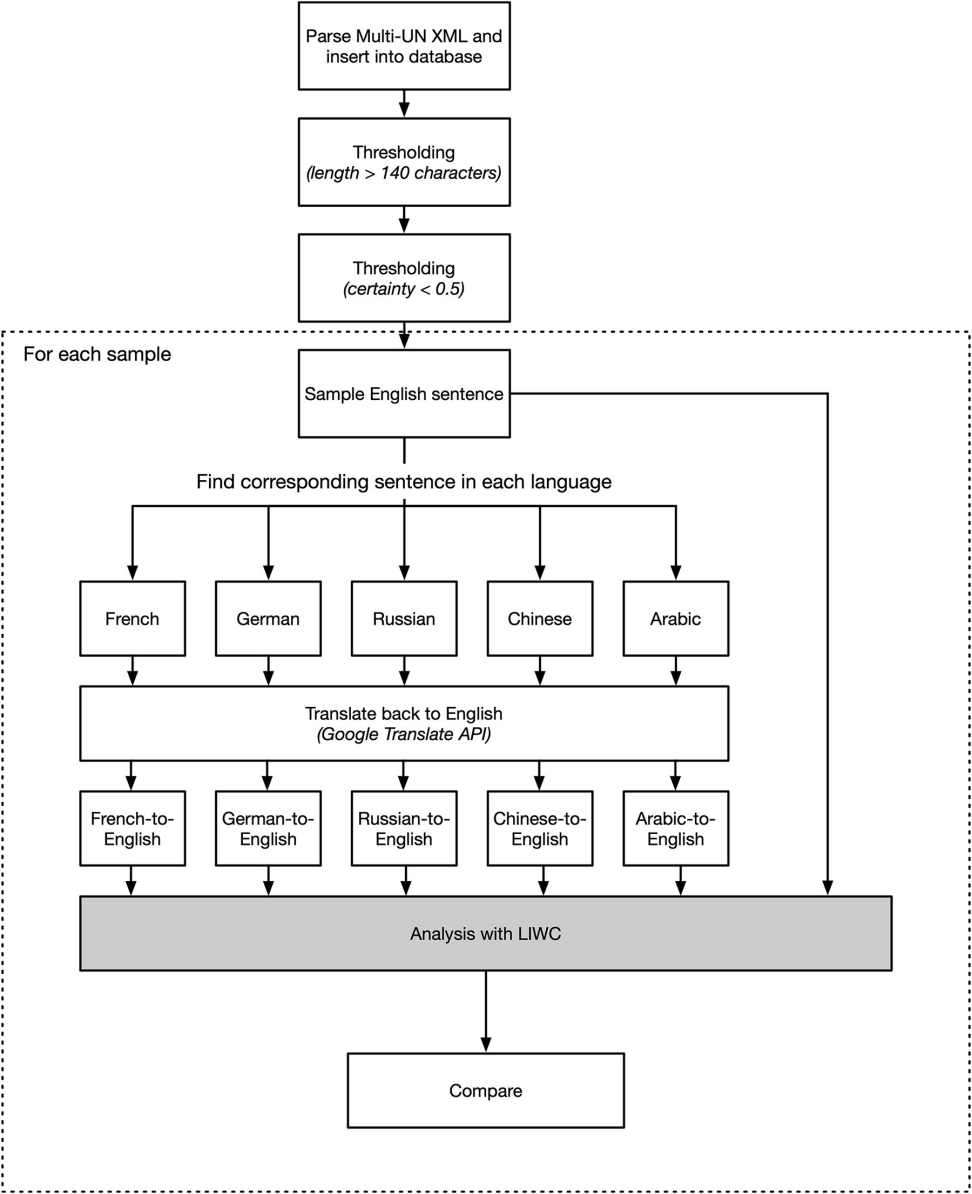
Workflow.

Using this method, we sampled over 3,000 parallel sentences. The next step was to produce machine translations for each individual sentence. Using the Google Translate API, each individual sentence in a parallel sentence set was translated to each of the other five languages. This process was performed in an automated fashion, using a script written in Python with Kenneth Reitz’s *requests* library [47]. The result was a new set of parallel sentences with each member consisting of one English sentence taken directly from the corpus and five English sentences produce by machine translation of the five non-English language sentences from the corpus. Fig 2 exhibits the workflow in a flow chart.

### Analysis

We note that the unit of analysis here is the sentence, the minimal unit of analysis for which an assumption of independence is linguistically reasonable. Further, syntactic parsing is done at the sentence level [48]. Note that statistical tests involving LIWC proportions are sensitive to the unit taken, and best practice would be to segment all documents into sentences. This level of analysis using short utterances can be used to investigate broader social science research inquiry into formal genres of discourse the speeches of political leaders. Given that the LIWC program which we evaluate here is not grammatically-bound by rules of syntax but rather represents a “bag of words” approach, it may also be applicable to communication via venues such as Facebook posts and Twitter messages [49,50]. We note that while the lexicon of social media, including abbreviations, acronyms, urls, and hashtags may introduce noise into the data, the overarching LIWC categories should remain stable, These “bag of words” approaches to computational discourse analysis do not require lengthy texts, unlike tools that analyze syntax and cohesion that are dependent on longer documents [30,51].

After we removed sentences where we determined that the alignment had failed (removing sentences with fewer than 15 words or longer than 230 word, and sentences whose pairwise LSA matches with the aligned sentences was less than .4) we were left with a corpus of 3,447 sextuplets of English sentences (one originally in English and five produced from the other official UN languages by Google Translate). This comprises a total of 157,595 words in the English corpus. Each sentence was then analyzed using LIWC 2015. A table of the summary statistics from the Multi-UN corpus appears as S2 Table.

The primary goal of this paper is to quantify the effect of machine translation upon LIWC indices. Our primary measure is the correlation between the “original” English variable and the one computed from the machine translation (see S3 Table). We consider both the LIWC proportions and the associated raw LIWC word counts formed by rounding the product of the word count and the proportion (see S4 Table).

An alternative way of looking at the effect size is to look at confidence intervals for the mean difference and compare these with the mean for the English corpus (see S5 Table). This lets us compare the mean difference with the mean. The confidence intervals for the mean differences are computed using the t-distribution (though our sample size is sufficiently large that these are indistinguishable from the asymptotic normal distribution) and the sample standard deviation for the paired difference. Though the distributions of the LIWC indices themselves are highly asymmetric due to censoring, the paired difference is typically much more symmetric and unimodal and thus the central limit theorem is sufficient to make our confidence intervals reasonable. Closely related to the confidence intervals are t-tests for the paired differences (see S6 Table).

Finally, we compute a variant of the Cohen’s d effect size. The standard Cohen’s d for paired difference, useful for power analysis, is not useful for determining whether the difference is of practical importance since it normalizes with respect to the standard deviation of the difference. Instead, we use a variant of the Cohen’s d for independent samples, which normalizes the difference with respect to a measure of standard deviation for one or both of the variables. We choose to compute the variant that uses the scale of the English variable to normalize the difference. Thus, our effect size is given by

$$d=\frac{\mu_{\text{Translated}}-\mu_{\text{English}}}{\sigma_{\text{English}}}$$

A reasonable case could be made for using *σ*_Translated_ or the average $\sqrt{\frac{\sigma_{\text{English}}^{2}+\sigma_{\text{Translated}}^{2}}{2}}$ in the denominator but the effect is minimal and is, we felt, overweighed by the benefit of holding the denominator constant across the various comparisons.

We remark that, though it may be tempting to use statistical tests for proportions on the LIWC proportions this is not valid. LIWC proportions are proportions of total words and at the level of words no assumption of independence is valid.

## Results

Table 1 shows mean correlations of the variables in each of the categories (Summary Language Variables, Linguistic Dimensions, Other Grammar, and Psychological Processes, Punctuation) given in Table 1 of [33]. For a complete list of LIWC categories, indices, and example words, see S1 Table.

**Table 1:** Mean correlations of word proportions across LIWC categories and languages.

LIWC Category	Language translated from					
	Arabic	German	French	Russian	Mandarin	Mean
All	0.831	0.814	0.822	0.843	0.783	0.820
Summary	0.863	0.833	0.856	0.906	0.761	0.844
Linguistic Dim.	0.729	0.728	0.769	0.788	0.651	0.733
Other Grammar	0.829	0.784	0.783	0.856	0.724	0.795
Psych. Proc.	0.862	0.838	0.832	0.887	0.836	0.851
Punctuation	0.787	0.813	0.837	0.614	0.728	0.771

From this table we can see that the Summary Language Variables and the Psychological Processes categories have high mean correlation and Linguistic Dimensions the lowest correlations. Overall Russian is the most stable and Mandarin the least. Despite the lower correlations in other categories Mandarin still shows high correlation in the Psychological Processes category.

The Summary Language Variables are not proportions but the remaining categories are all reported as proportions. If we consider the associated word counts then then correlations are generally higher (75% of pairs have higher correlation), see Table 2.

**Table 2:** Mean correlations of word counts across LIWC categories and languages.

LIWC Category	Language translated from					
	Arabic	German	French	Russian	Mandarin	Mean
All	0.851	0.837	0.844	0.860	0.804	0.832
Summary	0.863	0.833	0.856	0.906	0.761	0.844
Linguistic Dim.	0.770	0.784	0.799	0.823	0.693	0.774
Other Grammar	0.848	0.791	0.804	0.867	0.754	0.813
Psychological Proc.	0.874	0.855	0.850	0.894	0.852	0.865
Punctuation	0.826	0.823	0.870	0.669	0.740	0.786

Summary is unchanged as its entries are not proportions and remain unchanged.

This indicates that in many cases the observed effect on the proportion is due to the uniform change in the word count, the denominator in the proportion, and not in the actual category word count, the numerator in the proportion. The authors prefer the use of word counts to word proportions.

Table 3 shows all the LIWC variables (proportions) whose mean correlation across the five languages is less than 0.8.

**Table 3:** LIWC variables with less than 0.8 mean correlation of word proportions.

Category	Variable
Composite	analytic
Linguistic Dimension	pronoun, ppron, we, you, shehe, they, ipron, prep, auxverb, adverb
Other Grammar	verb, compare, interrog
Psychological Processes	sad [Affective processes/Negative Emotions],male [Social processes], discrep [Cognitive processes], see [Perceptual processes], hear [Perceptual processes], reward [Drives], focuspast [Time orientation], focuspresent [Time orientation], focusfuture [Time orientation], motion [Relativity], home [Personal concerns], nonflu [Informal language]
Punctuation	Period, semic

When we look at the Psychological Processes, we see that every primary category variable is correlated at above 0.8. The category of time orientation (which has no associated primary variable) has all three of its constituent measures correlated at less than 0.8. Within the affective language category, the primary variable (affect) and the two secondary components (posemo, negemo) are correlated at better than 0.8. Switching from proportions to word counts would see pronoun, prep, reward, and focusfuture leave the list while percept and feel enter the list.

While we view the correlation and Cohen’s d effect size measurements as the most pertinent, we have also produced confidence intervals for the paired differences and their associated t-tests (see S5 and S6 Tables in the Appendix). We emphasize that a statistically significant difference may not be of practical significance. To determine whether the difference is of practical significance one could compare it with the standard deviation of the variable (which is what our Cohen’s d does) or, since the majority of the variables are normed, with the mean of the variable itself. S7 Table shows the confidence interval using percentages of the English variable mean. Our sample size is quite large, so our t-tests are sensitive to quite small changes. Given this is it somewhat remarkable the number of variables that do not show statistically significant changes.

We label our Cohen’s d effect sizes using a variation on Sawilowsky’s extension of Cohen’s original scheme [52,53]. In tests of interventions these are normally taken to be the lower limit of a range. Since we desire no effect from our intervention it seems reasonable to take these as the upper limit of a range instead, shown in Table 4 below.

**Table 4:** Interpretation of effect sizes.

*d*	Interpretation
0 ≤ |*d*| < 0.01	Very small
0.01 ≤ |*d*| < 0.2	Small
0.2 ≤ |*d*| < 0.5	Medium
0.5 ≤ |*d*| < 0.8	Large

A complete table of Cohen’s d effect sizes is given in S8 Table. None of our Cohen’s d effect sizes exceeds 0.8 in magnitude. Most encouragingly, with one exception (time in Arabic) all of the Psychological Processes, and all the new LIWC 2015 composite indices, exhibit effect sizes termed either “very small” or “small”. Effect sizes termed “large” are confined to Mandarin. Indices with an effect size categorized as “medium'' or larger are summarized in Table 5 below. Russian is not included, as no variable pairings exhibited anything more than a “small” effect size.

**Table 5:** LIWC variables showing medium or greater effect size.

LIWC Variable	Language Translated From			
	Arabic	German	French	Chinese
wps	0.135	-0.409	-0.01	-0.586
dic	-0.014	-0.036	-0.016	-0.305
function	-0.05	0.049	0.095	-0.598
pronoun	-0.015	0.127	0.345	-0.257
you	0.287	0.105	0	0.089
ipron	-0.01	0.099	0.33	-0.238
prep	-0.117	-0.131	-0.124	-0.701
negate	0.002	-0.039	0.031	0.541
time	0.276	0	0.042	0.079
Effect Size	Very Small	Small	Medium	Large

To produce an overall effect size we treated the various LIWC variables as independent (we exclude the category variables and only consider the subcategorical variables) and sum the differences and the variances of the English variables.

## Conclusions

Text-as-data research in political science usually employs English-language corpora, even in international relations research. To internationalize this line of inquiry, our findings suggest that Google Translate may help scholars to overcome the deficit of non-English corpora. Human translation is time-consuming and expensive; machine translation, on the other hand, is quick and less costly.

To summarize, the LIWC indices do change under translation but in most cases the change is small relative to the standard deviation of the variable and as a percentage of the mean value of the variable in the English corpus. The Linguistic Dimensions and Other Grammar parts of LIWC output are the most unstable under translation and care should be exercised when trying to use these in analyses involving translated documents. Fortunately, many of the popular Psychological Processes, including all the core categories are very stable under translation.

While much of the computational analysis of political language has moved beyond using simple word count tools like LIWC [22,45], this linguistics tool continues to reliably provide valuable information about textual corpora [18]. In future work, we intend to explore this issue of translation stability in several ways. First, we have identified a corpus with longer documents (instead of single sentences) using expert human translation [54]. We have translated these from German to English using Google Translate and are in the process of aligning and analyzing the corpus. Second, we intend to replicate the translation stability verification process natural language processing (NLP) programs that analyzes semantics and syntax. To validate the full suite of indices, this program requires longer blocks of text than single sentences as it measures both referential (sentence-to-sentence) and deep (document-level) cohesion. This process of translation, analysis, and validation can be replicated with a suite of other computational tools as well [31,55].

One issue that any bag-of-words analysis falls short in addressing, is that of translating words versus translating meaning. Meanings of words vary across language, culture, and time; it is not clear whether direct translation via Google Translate will accurately capture the intended meaning from one actor to another. Issues of polysemy–the simultaneous existence of multiple interpretations of words and phrases–cannot always be captured by bag-of-words approaches. This requires more computationally intensive programs that examine the syntactic and semantic properties of language; such a project is currently under development for English, Chinese, French, Spanish, German, Arabic, and Russian [56].

Overall, we are encouraged by our findings from this study of expert human and computer translations. We acknowledge potential endogeneity concerns given that Google drew linguistic information from United Nations documents; however, the MultiUN corpus represents only a tiny proportion of the total number of parallel documents presumably used in generating statistical machine translation algorithms, and our analyzed subset is smaller still. Given the preponderance of questions that scholars receive about the validity of linguistic measures across translated documents, we believe that this study will help to assuage concerns about the generalizability of findings. Further, we have demonstrated that Google Translate is a reliable venue for accurate automated document translation. In summary, typically, the more frequent the word category the more stable it was under translation. Thus, if you observe a medium or large effect size change in a category that appears frequently then it is unlikely to have arisen purely as an artifact of translation.

## Supporting information

S1 Table LIWC categories, indices, and examples. (DOCX)

S2 Table Summary statistics for LIWC variables (proportions) across languages. (DOCX)

S3 Table Correlations between LIWC variables (proportions) on English sentence and on machine translated sentence. (DOCX)

S4 Table Correlations between LIWC variables (word counts) on English sentence and on machine translated sentence. (DOCX)

S5 Table Confidence intervals for the paired difference of LIWC variables (proportions) across languages. (DOCX)

S6 Table p-Values for t-test of paired differences from English. (DOCX)

S7 Table Confidence intervals for the paired difference of LIWC variables (proportions) across languages expressed as percentages of the English mean. (DOCX)

S8 Table Cohen’s d effect sizes across languages. (DOCX)
