# Supplementary material for: Automated content analysis across six languages
Source: PLoS One. 2019 Nov 20;14(11):e0224425. doi: 10.1371/journal.pone.0224425 (PMC6867602; doi:10.1371/journal.pone.0224425)
Supplement: S1 Table — (DOCX) [file pone.0224425.s001.docx]

S1 Table: LIWC categories, indices, and examples

| **Category** | **Abbrev** | **Examples** |
| --- | --- | --- |
| Linguistic Processes |  |  |
| Word count | wc |  |
| words/sentence | wps |  |
| Dictionary words | dic |  |
| Words>6 letters | sixltr |  |
| Total function words | funct |  |
| Total pronouns | pronoun | I, them, itself |
| Personal pronouns | ppron | I, them, her |
| 1st pers singular | i | I, me, mine |
| 1st pers plural | we | We, us, our |
| 2nd person | you | You, your, thou |
| 3rd pers singular | shehe | She, her, him |
| 3rd pers plural | they | They, their, they’d |
| Impersonal pronouns | ipron | It, it’s, those |
| Articles | article | A, an, the |
| Common verbs | verb | Walk, went, see |
| Auxiliary verbs | auxverb | Am, will, have |
| Past tense | past | Went, ran, had |
| Present tense | present | Is, does, hear |
| Future tense | future | Will, gonna |
| Adverbs | adverb | Very, really, quickly |
| Prepositions | prep | To, with, above |
| Conjunctions | conj | And, but, whereas |
| Negations | negate | No, not, never |
| Quantifiers | quant | Few, many, much |
| Numbers | number | Second, thousand |
| Swear words | swear | Damn, piss, fuck |
| Psychological Processes |  |  |
| Social processes | social | Mate, talk, they, child |
| Family | family | Daughter, husband, aunt |
| Friends | friend | Buddy, friend, neighbor |
| Humans | human | Adult, baby, boy |
| Affective processes | affect | Happy, cried, abandon |
| Positive emotion | posemo | Love, nice, sweet |
| Negative emotion | negemo | Hurt, ugly, nasty |
| Anxiety | anx | Worried, fearful, nervous |
| Anger | anger | Hate, kill, annoyed |
| Sadness | sad | Crying, grief, sad |
| Cognitive processes | cogmech | cause, know, ought |
| Insight | insight | think, know, consider |
| Causation | cause | because, effect, hence |
| Discrepancy | discrep | should, would, could |
| Tentative | tentat | maybe, perhaps, guess |
| Certainty | certain | always, never |
| Inhibition | inhib | block, constrain, stop |
| Inclusive | incl | And, with, include |
| Exclusive | excl | But, without, exclude |
| Perceptual processes | percept | Observing, heard, feeling |
| See | see | View, saw, seen |
| Hear | hear | Listen, hearing |
| Feel | feel | Feels, touch |
| Biological processes | bio | Eat, blood, pain |
| Body | body | Cheek, hands, spit |
| Health | health | Clinic, flu, pill |
| Sexual | sexual | Horny, love, incest |
| Ingestion | ingest | Dish, eat, pizza |
| Relativity | relativ | Area, bend, exit, stop |
| Motion | motion | Arrive, car, go |
| Space | space | Down, in, thin |
| Time | time | End, until, season |
| Personal Concerns |  |  |
| Work | work | Job, majors, xerox |
| Achievement | achieve | Earn, hero, win |
| Leisure | leisure | Cook, chat, movie |
| Home | home | Apartment, kitchen, family |
| Money | money | Audit, cash, owe |
| Religion | relig | Altar, church, mosque |
| Death | death | Bury, coffin, kill |
| Spoken categories |  |  |
| Assent | assent | Agree, OK, yes |
| Nonfluencies | nonflu | Er, hm, umm |
| Fillers | filler | Blah, Imean, youknow |
| Composite |  |  |
| Power |  |  |
| Clout |  |  |
| Affiliation |  |  |
| Analytic |  |  |
| Authentic |  |  |
| Emotional Tone |  |  |
