# Supplementary material for: Automated content analysis across six languages
Source: PLoS One. 2019 Nov 20;14(11):e0224425. doi: 10.1371/journal.pone.0224425 (PMC6867602; doi:10.1371/journal.pone.0224425)
Supplement: S2 Table — (DOCX) [file pone.0224425.s002.docx]

S2 Table: Summary statistics for LIWC variables (proportions) across languages

|  |  |  | Language Translated From | | | | | | | | | |
| --- | --- | --- | --- | --- | --- | --- | --- | --- | --- | --- | --- | --- |
| LIWC Variable | English | | Arabic | | German | | French | | Russian | | Mandarin | |
|  | Mean | Std. Dev. | Mean | Std. Dev. | Mean | Std. Dev. | Mean | Std. Dev. | Mean | Std. Dev. | Mean | Std. Dev. |
| wc | 45.72 | 21.96 | 48.96 | 24.03 | 46.56 | 22.38 | 45.92 | 22.22 | 45.84 | 21.65 | 44.76 | 21.82 |
| analytic | 97.24 | 5.88 | 97.12 | 5.81 | 97.05 | 6.22 | 96.51 | 7.43 | 97.39 | 5.63 | 96.19 | 6.98 |
| clout | 64.06 | 15.72 | 63.95 | 15.23 | 64.02 | 15.60 | 63.06 | 15.88 | 64.30 | 15.61 | 62.88 | 17.92 |
| authentic | 32.31 | 26.92 | 36.03 | 29.61 | 30.64 | 26.81 | 29.95 | 26.19 | 32.92 | 27.37 | 31.26 | 28.10 |
| tone | 56.20 | 34.74 | 55.25 | 34.15 | 56.08 | 34.37 | 54.63 | 34.53 | 56.14 | 34.55 | 56.32 | 35.18 |
| wps | 45.01 | 22.46 | 48.05 | 24.15 | 35.82 | 18.65 | 44.80 | 22.67 | 45.56 | 21.80 | 31.85 | 16.80 |
| sixltr | 34.65 | 8.72 | 33.59 | 7.85 | 33.05 | 8.31 | 33.08 | 8.42 | 34.02 | 8.38 | 34.81 | 8.51 |
| dic | 73.60 | 11.30 | 73.44 | 9.99 | 73.19 | 11.36 | 73.42 | 11.32 | 73.18 | 11.77 | 70.15 | 12.23 |
| function | 40.46 | 7.81 | 40.06 | 7.37 | 40.84 | 7.89 | 41.20 | 7.90 | 40.39 | 8.00 | 35.79 | 8.29 |
| pronoun | 3.61 | 3.14 | 3.56 | 3.06 | 4.01 | 3.25 | 4.69 | 3.78 | 3.93 | 3.20 | 2.80 | 2.65 |
| ppron | 0.59 | 1.39 | 0.57 | 1.31 | 0.70 | 1.51 | 0.71 | 1.53 | 0.67 | 1.42 | 0.48 | 1.17 |
| i | 0.11 | 0.73 | 0.10 | 0.65 | 0.11 | 0.74 | 0.11 | 0.69 | 0.12 | 0.76 | 0.08 | 0.55 |
| we | 0.01 | 0.25 | 0.02 | 0.30 | 0.02 | 0.28 | 0.03 | 0.33 | 0.03 | 0.34 | 0.05 | 0.39 |
| you | 0.00 | 0.02 | 0.01 | 0.15 | 0.00 | 0.08 | 0.00 | 0.02 | 0.00 | 0.05 | 0.00 | 0.07 |
| shehe | 0.07 | 0.50 | 0.10 | 0.50 | 0.11 | 0.58 | 0.13 | 0.62 | 0.10 | 0.52 | 0.08 | 0.47 |
| they | 0.39 | 1.09 | 0.34 | 1.00 | 0.46 | 1.19 | 0.44 | 1.22 | 0.41 | 1.08 | 0.27 | 0.86 |
| ipron | 3.02 | 2.92 | 2.99 | 2.81 | 3.31 | 2.92 | 3.98 | 3.45 | 3.26 | 2.93 | 2.33 | 2.45 |
| article | 10.62 | 5.03 | 10.71 | 4.74 | 10.96 | 5.05 | 10.46 | 4.92 | 10.67 | 4.85 | 9.93 | 4.90 |
| prep | 19.02 | 5.00 | 18.44 | 4.97 | 18.36 | 4.76 | 18.40 | 4.92 | 18.89 | 4.99 | 15.52 | 5.67 |
| auxverb | 1.36 | 2.33 | 1.13 | 2.03 | 1.76 | 2.45 | 1.78 | 2.49 | 1.20 | 2.18 | 1.33 | 2.22 |
| adverb | 0.92 | 1.58 | 0.89 | 1.52 | 0.90 | 1.62 | 0.92 | 1.60 | 0.92 | 1.59 | 0.95 | 1.62 |
| conj | 5.75 | 3.72 | 6.06 | 3.90 | 5.48 | 3.66 | 5.52 | 3.59 | 5.61 | 3.66 | 5.47 | 3.71 |
| negate | 0.23 | 0.83 | 0.23 | 0.80 | 0.20 | 0.77 | 0.26 | 0.89 | 0.18 | 0.71 | 0.68 | 1.79 |
| verb | 3.70 | 3.43 | 3.64 | 3.20 | 4.13 | 3.52 | 4.25 | 3.70 | 3.51 | 3.32 | 3.69 | 3.48 |
| adj | 3.90 | 3.37 | 3.55 | 3.15 | 3.62 | 3.28 | 3.36 | 3.18 | 3.89 | 3.33 | 3.79 | 3.43 |
| compare | 1.36 | 2.05 | 1.16 | 1.87 | 1.18 | 1.94 | 1.14 | 1.91 | 1.37 | 2.06 | 1.22 | 2.03 |
| interrog | 0.37 | 1.01 | 0.41 | 1.01 | 0.46 | 1.07 | 0.44 | 1.04 | 0.41 | 1.02 | 0.23 | 0.79 |
| number | 8.84 | 12.47 | 9.48 | 10.82 | 9.67 | 12.37 | 9.08 | 12.45 | 8.87 | 12.42 | 10.55 | 12.61 |
| quant | 1.06 | 1.80 | 1.10 | 1.81 | 1.12 | 1.84 | 1.17 | 1.89 | 1.14 | 1.90 | 1.14 | 1.90 |
| affect | 4.19 | 4.22 | 4.03 | 4.02 | 4.13 | 4.09 | 4.11 | 4.14 | 4.17 | 4.18 | 4.37 | 4.41 |
| posemo | 3.15 | 3.37 | 3.00 | 3.18 | 3.09 | 3.25 | 3.01 | 3.23 | 3.14 | 3.32 | 3.26 | 3.50 |
| negemo | 0.98 | 2.20 | 0.96 | 2.13 | 0.98 | 2.17 | 1.03 | 2.24 | 0.98 | 2.22 | 1.05 | 2.36 |
| anx | 0.27 | 0.96 | 0.28 | 0.93 | 0.28 | 0.96 | 0.30 | 0.99 | 0.27 | 0.94 | 0.30 | 1.01 |
| anger | 0.39 | 1.26 | 0.39 | 1.27 | 0.38 | 1.23 | 0.40 | 1.26 | 0.39 | 1.26 | 0.40 | 1.35 |
| sad | 0.07 | 0.52 | 0.07 | 0.50 | 0.06 | 0.45 | 0.07 | 0.47 | 0.07 | 0.48 | 0.07 | 0.50 |
| social | 5.17 | 4.29 | 5.02 | 4.14 | 5.11 | 4.21 | 5.00 | 4.28 | 5.19 | 4.32 | 5.29 | 4.32 |
| family | 0.02 | 0.26 | 0.02 | 0.25 | 0.03 | 0.31 | 0.02 | 0.26 | 0.02 | 0.24 | 0.02 | 0.29 |
| friend | 0.12 | 0.59 | 0.09 | 0.51 | 0.09 | 0.51 | 0.13 | 0.61 | 0.10 | 0.53 | 0.11 | 0.54 |
| female | 0.09 | 0.60 | 0.09 | 0.59 | 0.10 | 0.61 | 0.09 | 0.57 | 0.10 | 0.60 | 0.10 | 0.61 |
| male | 0.12 | 0.60 | 0.12 | 0.58 | 0.13 | 0.63 | 0.23 | 0.82 | 0.13 | 0.61 | 0.10 | 0.52 |
| cogproc | 6.92 | 4.69 | 6.77 | 4.59 | 6.89 | 4.66 | 7.16 | 4.77 | 6.97 | 4.64 | 7.50 | 5.10 |
| insight | 3.32 | 3.18 | 3.16 | 2.98 | 3.26 | 3.15 | 3.30 | 3.16 | 3.29 | 3.13 | 3.61 | 3.68 |
| cause | 1.33 | 2.00 | 1.30 | 1.93 | 1.30 | 1.94 | 1.31 | 2.00 | 1.33 | 1.99 | 1.43 | 2.13 |
| discrep | 0.38 | 1.04 | 0.38 | 1.01 | 0.39 | 1.07 | 0.44 | 1.12 | 0.42 | 1.09 | 0.48 | 1.18 |
| tentat | 0.71 | 1.68 | 0.76 | 1.82 | 0.71 | 1.56 | 0.72 | 1.64 | 0.69 | 1.62 | 0.72 | 1.68 |
| certain | 0.96 | 1.74 | 0.96 | 1.69 | 1.05 | 1.81 | 1.13 | 1.94 | 0.99 | 1.74 | 0.99 | 1.75 |
| differ | 0.80 | 1.76 | 0.75 | 1.71 | 0.80 | 1.75 | 0.92 | 1.86 | 0.80 | 1.76 | 0.85 | 1.86 |
| percept | 0.30 | 1.02 | 0.29 | 0.99 | 0.29 | 0.98 | 0.28 | 1.00 | 0.22 | 0.82 | 0.29 | 1.02 |
| see | 0.22 | 0.83 | 0.20 | 0.79 | 0.20 | 0.78 | 0.17 | 0.73 | 0.14 | 0.61 | 0.20 | 0.80 |
| hear | 0.03 | 0.29 | 0.03 | 0.28 | 0.04 | 0.36 | 0.04 | 0.37 | 0.03 | 0.32 | 0.04 | 0.34 |
| feel | 0.04 | 0.38 | 0.05 | 0.39 | 0.04 | 0.37 | 0.07 | 0.47 | 0.04 | 0.39 | 0.05 | 0.40 |
| bio | 0.65 | 1.59 | 0.64 | 1.62 | 0.62 | 1.57 | 0.64 | 1.56 | 0.64 | 1.59 | 0.61 | 1.61 |
| body | 0.15 | 0.65 | 0.16 | 0.65 | 0.13 | 0.59 | 0.15 | 0.64 | 0.15 | 0.63 | 0.15 | 0.66 |
| health | 0.33 | 1.27 | 0.32 | 1.32 | 0.32 | 1.22 | 0.35 | 1.26 | 0.32 | 1.26 | 0.32 | 1.30 |
| sexual | 0.07 | 0.60 | 0.05 | 0.48 | 0.07 | 0.62 | 0.06 | 0.58 | 0.06 | 0.58 | 0.07 | 0.66 |
| ingest | 0.14 | 0.68 | 0.14 | 0.64 | 0.15 | 0.74 | 0.12 | 0.63 | 0.15 | 0.68 | 0.12 | 0.63 |
| drives | 7.63 | 5.34 | 7.62 | 5.23 | 7.65 | 5.26 | 7.50 | 5.29 | 7.57 | 5.30 | 8.05 | 5.56 |
| affiliation | 1.51 | 2.17 | 1.50 | 2.10 | 1.47 | 2.10 | 1.57 | 2.21 | 1.48 | 2.12 | 1.64 | 2.32 |
| achieve | 1.65 | 2.41 | 1.72 | 2.43 | 1.66 | 2.37 | 1.59 | 2.37 | 1.67 | 2.43 | 1.85 | 2.56 |
| power | 3.10 | 3.18 | 3.07 | 3.03 | 3.15 | 3.15 | 3.20 | 3.20 | 3.03 | 3.19 | 3.18 | 3.24 |
| reward | 0.80 | 1.51 | 0.90 | 1.60 | 0.90 | 1.58 | 0.83 | 1.57 | 0.86 | 1.57 | 0.85 | 1.66 |
| risk | 1.14 | 2.07 | 1.08 | 1.95 | 1.11 | 2.02 | 0.96 | 1.85 | 1.14 | 2.05 | 1.20 | 2.15 |
| focuspast | 0.81 | 1.52 | 0.84 | 1.49 | 0.91 | 1.56 | 0.95 | 1.68 | 0.84 | 1.53 | 0.96 | 1.68 |
| focuspresent | 2.61 | 2.84 | 2.57 | 2.80 | 2.93 | 2.94 | 3.01 | 3.04 | 2.43 | 2.78 | 2.58 | 2.83 |
| focusfuture | 0.52 | 1.25 | 0.49 | 1.28 | 0.50 | 1.22 | 0.48 | 1.18 | 0.47 | 1.18 | 0.51 | 1.24 |
| relativ | 13.26 | 5.43 | 14.29 | 5.99 | 12.99 | 5.44 | 12.70 | 5.38 | 13.48 | 5.60 | 12.72 | 5.72 |
| motion | 0.69 | 1.39 | 0.73 | 1.37 | 0.74 | 1.41 | 0.67 | 1.33 | 0.65 | 1.33 | 0.70 | 1.37 |
| space | 8.92 | 5.01 | 8.80 | 4.87 | 8.63 | 4.97 | 8.25 | 4.78 | 9.10 | 5.08 | 8.06 | 4.93 |
| time | 3.60 | 3.85 | 4.66 | 5.42 | 3.60 | 3.83 | 3.76 | 3.95 | 3.64 | 3.95 | 3.90 | 4.21 |
| work | 6.98 | 5.11 | 6.75 | 4.97 | 6.80 | 4.96 | 6.80 | 5.07 | 6.86 | 5.04 | 7.35 | 5.32 |
| leisure | 0.36 | 1.03 | 0.36 | 1.01 | 0.37 | 1.06 | 0.35 | 1.02 | 0.36 | 1.03 | 0.35 | 1.03 |
| home | 0.07 | 0.47 | 0.07 | 0.45 | 0.06 | 0.41 | 0.07 | 0.47 | 0.06 | 0.42 | 0.08 | 0.50 |
| money | 1.07 | 2.12 | 1.00 | 1.94 | 0.97 | 1.94 | 1.08 | 2.12 | 1.02 | 1.99 | 1.08 | 2.11 |
| relig | 0.11 | 0.65 | 0.10 | 0.64 | 0.10 | 0.62 | 0.11 | 0.64 | 0.10 | 0.62 | 0.11 | 0.69 |
| death | 0.08 | 0.52 | 0.06 | 0.42 | 0.07 | 0.49 | 0.07 | 0.47 | 0.07 | 0.49 | 0.08 | 0.52 |
| informal | 0.27 | 0.87 | 0.24 | 0.79 | 0.24 | 0.82 | 0.22 | 0.81 | 0.30 | 0.90 | 0.26 | 0.86 |
| swear | 0.00 | 0.06 | 0.00 | 0.05 | 0.00 | 0.06 | 0.00 | 0.06 | 0.00 | 0.06 | 0.00 | 0.06 |
| netspeak | 0.14 | 0.69 | 0.13 | 0.63 | 0.15 | 0.71 | 0.15 | 0.71 | 0.14 | 0.70 | 0.16 | 0.72 |
| assent | 0.01 | 0.18 | 0.01 | 0.20 | 0.01 | 0.18 | 0.01 | 0.15 | 0.01 | 0.18 | 0.01 | 0.18 |
| nonflu | 0.12 | 0.51 | 0.09 | 0.44 | 0.08 | 0.40 | 0.07 | 0.38 | 0.14 | 0.56 | 0.09 | 0.44 |
| filler | . | . | . | . | . | . | . | . | . | . | . | . |
| allpunc | 14.63 | 10.10 | 17.78 | 10.51 | 14.19 | 9.94 | 14.55 | 10.39 | 12.36 | 10.26 | 20.73 | 12.80 |
| period | 0.94 | 2.31 | 0.82 | 1.97 | 1.80 | 2.38 | 0.94 | 2.35 | 0.12 | 0.82 | 2.23 | 2.68 |
| comma | 6.34 | 4.87 | 6.05 | 4.53 | 5.30 | 4.50 | 5.80 | 4.64 | 5.32 | 4.67 | 7.30 | 5.38 |
| colon | 0.13 | 0.58 | 0.13 | 0.58 | 0.13 | 0.59 | 0.14 | 0.62 | 0.05 | 0.36 | 0.17 | 0.68 |
| semic | 0.92 | 1.38 | 1.79 | 2.18 | 1.43 | 1.96 | 1.79 | 2.18 | 0.85 | 2.07 | 3.13 | 3.44 |
| qmark | . | . | . | . | . | . | . | . | . | . | . | . |
| exclam | . | . | . | . | . | . | . | . | . | . | . | . |
| dash | 1.48 | 2.19 | 2.20 | 2.53 | 1.12 | 1.88 | 1.08 | 1.82 | 1.39 | 2.06 | 1.40 | 2.20 |
| quote | 0.80 | 2.01 | . | . | . | . | . | . | 0.00 | 0.11 | . | . |
| apostro | 0.17 | 0.67 | . | . | . | . | . | . | . | . | . | . |
| parenth | 2.33 | 5.00 | 2.99 | 5.03 | 2.18 | 4.86 | 2.16 | 4.82 | 2.06 | 4.70 | 2.44 | 5.06 |
| otherp | 1.52 | 3.77 | 3.81 | 5.26 | 2.22 | 4.08 | 2.65 | 4.32 | 2.55 | 4.39 | 4.07 | 5.11 |
