# Supplementary material for: Automated content analysis across six languages
Source: PLoS One. 2019 Nov 20;14(11):e0224425. doi: 10.1371/journal.pone.0224425 (PMC6867602; doi:10.1371/journal.pone.0224425)
Supplement: S3 Table — (DOCX) [file pone.0224425.s003.docx]

S3 Table: Correlations between LIWC variables (proportions) on English sentence and on machine translated sentence.

| LIWC Variable | Language Translated From | | | | |  |
| --- | --- | --- | --- | --- | --- | --- |
|  | Arabic | German | French | Russian | Mandarin | Mean |
| wc | 0.963 | 0.970 | 0.978 | 0.945 | 0.972 | 0.966 |
| analytic | 0.693 | 0.723 | 0.698 | 0.855 | 0.497 | 0.693 |
| clout | 0.907 | 0.884 | 0.879 | 0.916 | 0.843 | 0.886 |
| authentic | 0.809 | 0.832 | 0.809 | 0.875 | 0.771 | 0.819 |
| tone | 0.908 | 0.878 | 0.847 | 0.909 | 0.885 | 0.885 |
| wps | 0.948 | 0.720 | 0.964 | 0.930 | 0.579 | 0.828 |
| sixltr | 0.865 | 0.868 | 0.864 | 0.909 | 0.841 | 0.870 |
| dic | 0.912 | 0.928 | 0.931 | 0.950 | 0.910 | 0.926 |
| function | 0.845 | 0.868 | 0.862 | 0.897 | 0.809 | 0.856 |
| pronoun | 0.791 | 0.805 | 0.695 | 0.848 | 0.638 | 0.755 |
| ppron | 0.717 | 0.724 | 0.725 | 0.827 | 0.720 | 0.742 |
| i | 0.908 | 0.935 | 0.963 | 0.951 | 0.881 | 0.928 |
| we | 0.786 | 0.733 | 0.769 | 0.624 | 0.630 | 0.709 |
| you | 0.138 | 0.294 | 1.000 | 0.535 | 0.293 | 0.452 |
| shehe | 0.514 | 0.406 | 0.338 | 0.492 | 0.511 | 0.452 |
| they | 0.673 | 0.706 | 0.736 | 0.822 | 0.724 | 0.732 |
| ipron | 0.780 | 0.783 | 0.680 | 0.835 | 0.623 | 0.740 |
| article | 0.843 | 0.859 | 0.842 | 0.894 | 0.776 | 0.843 |
| prep | 0.831 | 0.797 | 0.776 | 0.850 | 0.675 | 0.786 |
| auxverb | 0.670 | 0.680 | 0.698 | 0.806 | 0.625 | 0.696 |
| adverb | 0.736 | 0.599 | 0.652 | 0.793 | 0.603 | 0.677 |
| conj | 0.809 | 0.902 | 0.903 | 0.943 | 0.865 | 0.885 |
| negate | 0.898 | 0.825 | 0.892 | 0.703 | 0.385 | 0.741 |
| verb | 0.756 | 0.722 | 0.709 | 0.825 | 0.685 | 0.739 |
| adj | 0.877 | 0.829 | 0.782 | 0.890 | 0.785 | 0.833 |
| compare | 0.815 | 0.695 | 0.675 | 0.804 | 0.624 | 0.723 |
| interrog | 0.658 | 0.595 | 0.663 | 0.742 | 0.474 | 0.626 |
| number | 0.980 | 0.987 | 0.994 | 0.993 | 0.981 | 0.987 |
| quant | 0.888 | 0.878 | 0.876 | 0.881 | 0.794 | 0.863 |
| affect | 0.932 | 0.913 | 0.902 | 0.937 | 0.924 | 0.922 |
| posemo | 0.932 | 0.897 | 0.878 | 0.932 | 0.918 | 0.911 |
| negemo | 0.917 | 0.928 | 0.914 | 0.933 | 0.920 | 0.922 |
| anx | 0.826 | 0.873 | 0.829 | 0.894 | 0.858 | 0.856 |
| anger | 0.929 | 0.914 | 0.898 | 0.932 | 0.918 | 0.918 |
| sad | 0.739 | 0.799 | 0.770 | 0.821 | 0.805 | 0.787 |
| social | 0.910 | 0.871 | 0.884 | 0.918 | 0.887 | 0.894 |
| family | 0.989 | 0.842 | 0.995 | 0.955 | 0.985 | 0.953 |
| friend | 0.834 | 0.745 | 0.918 | 0.830 | 0.764 | 0.818 |
| female | 0.914 | 0.829 | 0.871 | 0.900 | 0.948 | 0.892 |
| male | 0.624 | 0.492 | 0.560 | 0.610 | 0.564 | 0.570 |
| cogproc | 0.896 | 0.868 | 0.870 | 0.915 | 0.852 | 0.880 |
| insight | 0.929 | 0.903 | 0.903 | 0.934 | 0.868 | 0.907 |
| cause | 0.854 | 0.793 | 0.811 | 0.858 | 0.818 | 0.827 |
| discrep | 0.800 | 0.776 | 0.713 | 0.854 | 0.688 | 0.766 |
| tentat | 0.879 | 0.848 | 0.850 | 0.916 | 0.862 | 0.871 |
| certain | 0.861 | 0.841 | 0.816 | 0.913 | 0.829 | 0.852 |
| differ | 0.899 | 0.897 | 0.860 | 0.924 | 0.890 | 0.894 |
| percept | 0.859 | 0.840 | 0.753 | 0.738 | 0.813 | 0.801 |
| see | 0.859 | 0.850 | 0.796 | 0.650 | 0.828 | 0.796 |
| hear | 0.810 | 0.723 | 0.589 | 0.854 | 0.622 | 0.720 |
| feel | 0.813 | 0.853 | 0.672 | 0.896 | 0.851 | 0.817 |
| bio | 0.889 | 0.895 | 0.895 | 0.945 | 0.905 | 0.906 |
| body | 0.812 | 0.855 | 0.820 | 0.929 | 0.869 | 0.857 |
| health | 0.898 | 0.902 | 0.924 | 0.949 | 0.935 | 0.921 |
| sexual | 0.895 | 0.961 | 0.976 | 0.977 | 0.980 | 0.958 |
| ingest | 0.928 | 0.894 | 0.840 | 0.942 | 0.830 | 0.887 |
| drives | 0.923 | 0.908 | 0.897 | 0.928 | 0.905 | 0.912 |
| affiliation | 0.929 | 0.898 | 0.909 | 0.914 | 0.913 | 0.912 |
| achieve | 0.876 | 0.867 | 0.849 | 0.900 | 0.858 | 0.870 |
| power | 0.889 | 0.870 | 0.879 | 0.920 | 0.891 | 0.890 |
| reward | 0.824 | 0.786 | 0.728 | 0.823 | 0.759 | 0.784 |
| risk | 0.947 | 0.937 | 0.894 | 0.953 | 0.934 | 0.933 |
| focuspast | 0.651 | 0.623 | 0.683 | 0.763 | 0.555 | 0.655 |
| focuspresent | 0.814 | 0.779 | 0.765 | 0.834 | 0.695 | 0.777 |
| focusfuture | 0.768 | 0.786 | 0.791 | 0.872 | 0.747 | 0.793 |
| relativ | 0.812 | 0.840 | 0.826 | 0.877 | 0.805 | 0.832 |
| motion | 0.770 | 0.713 | 0.691 | 0.774 | 0.780 | 0.746 |
| space | 0.874 | 0.855 | 0.842 | 0.896 | 0.807 | 0.855 |
| time | 0.920 | 0.928 | 0.935 | 0.950 | 0.930 | 0.933 |
| work | 0.935 | 0.918 | 0.920 | 0.947 | 0.923 | 0.929 |
| leisure | 0.909 | 0.869 | 0.838 | 0.918 | 0.850 | 0.877 |
| home | 0.805 | 0.763 | 0.762 | 0.833 | 0.816 | 0.796 |
| money | 0.932 | 0.911 | 0.944 | 0.920 | 0.933 | 0.928 |
| relig | 0.945 | 0.931 | 0.967 | 0.946 | 0.939 | 0.945 |
| death | 0.738 | 0.844 | 0.848 | 0.884 | 0.848 | 0.832 |
| informal | 0.864 | 0.780 | 0.781 | 0.861 | 0.739 | 0.805 |
| swear | 1.000 | 0.848 | 1.000 | 1.000 | 1.000 | 0.969 |
| netspeak | 0.954 | 0.959 | 0.961 | 0.936 | 0.916 | 0.945 |
| assent | 0.707 | 0.947 | 0.829 | 0.995 | 0.826 | 0.861 |
| nonflu | 0.724 | 0.372 | 0.377 | 0.727 | 0.356 | 0.511 |
| filler | . | . | . | . | . | . |
| allpunc | 0.870 | 0.899 | 0.906 | 0.911 | 0.837 | 0.885 |
| period | 0.948 | 0.785 | 0.975 | 0.624 | 0.635 | 0.793 |
| comma | 0.797 | 0.813 | 0.849 | 0.829 | 0.742 | 0.806 |
| colon | 0.868 | 0.855 | 0.959 | 0.595 | 0.858 | 0.827 |
| semic | 0.407 | 0.510 | 0.486 | -0.083 | 0.216 | 0.307 |
| qmark | . | . | . | . | . | . |
| exclam | . | . | . | . | . | . |
| dash | 0.753 | 0.815 | 0.749 | 0.826 | 0.874 | 0.803 |
| quote | . | . | . | 0.013 | . | . |
| apostro | . | . | . | . | . | . |
| parenth | 0.845 | 0.952 | 0.940 | 0.970 | 0.977 | 0.937 |
| otherp | 0.804 | 0.873 | 0.829 | 0.844 | 0.683 | 0.807 |
|  | 𝜌>.5 | 𝜌>.8 | 𝜌>.9 |  |  |  |
