# Supplementary material for: Automated content analysis across six languages
Source: PLoS One. 2019 Nov 20;14(11):e0224425. doi: 10.1371/journal.pone.0224425 (PMC6867602; doi:10.1371/journal.pone.0224425)
Supplement: S4 Table — (DOCX) [file pone.0224425.s004.docx]

S4 Table: Correlations between LIWC variables (word counts) on English sentence and on machine translated sentence.

| LIWC Variable | Language Translated From | | | | |  |
| --- | --- | --- | --- | --- | --- | --- |
|  | Arabic | German | French | Russian | Mandarin | Mean |
| sixltr | 0.942 | 0.963 | 0.966 | 0.959 | 0.960 | 0.958 |
| dic | 0.943 | 0.960 | 0.966 | 0.940 | 0.958 | 0.953 |
| function | 0.926 | 0.944 | 0.945 | 0.924 | 0.928 | 0.934 |
| pronoun | 0.842 | 0.848 | 0.760 | 0.866 | 0.711 | 0.805 |
| ppron | 0.731 | 0.736 | 0.715 | 0.816 | 0.727 | 0.745 |
| i | 0.919 | 0.944 | 0.945 | 0.954 | 0.890 | 0.930 |
| we | 0.729 | 0.751 | 0.712 | 0.677 | 0.533 | 0.681 |
| you | 0.392 | 0.577 | 1.000 | 0.816 | 0.577 | 0.672 |
| shehe | 0.545 | 0.446 | 0.420 | 0.526 | 0.493 | 0.486 |
| they | 0.725 | 0.743 | 0.728 | 0.809 | 0.748 | 0.751 |
| ipron | 0.830 | 0.824 | 0.732 | 0.862 | 0.678 | 0.785 |
| article | 0.879 | 0.898 | 0.884 | 0.891 | 0.835 | 0.878 |
| prep | 0.928 | 0.922 | 0.926 | 0.916 | 0.853 | 0.909 |
| auxverb | 0.678 | 0.704 | 0.720 | 0.803 | 0.651 | 0.711 |
| adverb | 0.740 | 0.635 | 0.667 | 0.811 | 0.606 | 0.692 |
| conj | 0.790 | 0.940 | 0.945 | 0.962 | 0.918 | 0.911 |
| negate | 0.901 | 0.850 | 0.890 | 0.710 | 0.249 | 0.720 |
| verb | 0.786 | 0.765 | 0.772 | 0.839 | 0.746 | 0.782 |
| adj | 0.896 | 0.841 | 0.816 | 0.903 | 0.818 | 0.855 |
| compare | 0.838 | 0.713 | 0.687 | 0.835 | 0.667 | 0.748 |
| interrog | 0.679 | 0.598 | 0.671 | 0.740 | 0.506 | 0.639 |
| number | 0.995 | 0.984 | 0.998 | 0.988 | 0.993 | 0.992 |
| quant | 0.892 | 0.844 | 0.881 | 0.897 | 0.794 | 0.862 |
| affect | 0.940 | 0.925 | 0.919 | 0.938 | 0.938 | 0.932 |
| posemo | 0.937 | 0.911 | 0.903 | 0.929 | 0.929 | 0.922 |
| negemo | 0.922 | 0.940 | 0.920 | 0.939 | 0.931 | 0.930 |
| anx | 0.844 | 0.869 | 0.851 | 0.906 | 0.876 | 0.869 |
| anger | 0.933 | 0.932 | 0.896 | 0.943 | 0.936 | 0.928 |
| sad | 0.734 | 0.787 | 0.774 | 0.809 | 0.732 | 0.767 |
| social | 0.924 | 0.897 | 0.900 | 0.929 | 0.905 | 0.911 |
| family | 1.000 | 0.742 | 1.000 | 0.913 | 0.975 | 0.926 |
| friend | 0.885 | 0.837 | 0.923 | 0.863 | 0.779 | 0.858 |
| female | 0.944 | 0.883 | 0.902 | 0.932 | 0.952 | 0.923 |
| male | 0.622 | 0.551 | 0.521 | 0.623 | 0.589 | 0.581 |
| cogproc | 0.915 | 0.906 | 0.907 | 0.930 | 0.888 | 0.909 |
| insight | 0.934 | 0.908 | 0.907 | 0.933 | 0.871 | 0.910 |
| cause | 0.857 | 0.808 | 0.829 | 0.866 | 0.830 | 0.838 |
| discrep | 0.810 | 0.795 | 0.749 | 0.860 | 0.707 | 0.784 |
| tentat | 0.904 | 0.889 | 0.881 | 0.940 | 0.898 | 0.903 |
| certain | 0.867 | 0.852 | 0.832 | 0.919 | 0.835 | 0.861 |
| differ | 0.904 | 0.914 | 0.881 | 0.930 | 0.905 | 0.907 |
| percept | 0.801 | 0.789 | 0.699 | 0.777 | 0.771 | 0.768 |
| see | 0.796 | 0.792 | 0.728 | 0.723 | 0.778 | 0.764 |
| hear | 0.778 | 0.696 | 0.653 | 0.838 | 0.657 | 0.724 |
| feel | 0.765 | 0.840 | 0.618 | 0.899 | 0.778 | 0.780 |
| bio | 0.897 | 0.903 | 0.911 | 0.942 | 0.924 | 0.915 |
| body | 0.851 | 0.846 | 0.819 | 0.915 | 0.885 | 0.863 |
| health | 0.904 | 0.913 | 0.938 | 0.951 | 0.951 | 0.931 |
| sexual | 0.935 | 0.974 | 0.985 | 0.983 | 0.976 | 0.970 |
| ingest | 0.913 | 0.893 | 0.828 | 0.914 | 0.828 | 0.875 |
| drives | 0.942 | 0.936 | 0.931 | 0.941 | 0.926 | 0.935 |
| affiliation | 0.932 | 0.910 | 0.919 | 0.922 | 0.921 | 0.921 |
| achieve | 0.888 | 0.891 | 0.872 | 0.908 | 0.831 | 0.878 |
| power | 0.914 | 0.903 | 0.905 | 0.924 | 0.912 | 0.911 |
| reward | 0.837 | 0.820 | 0.766 | 0.831 | 0.791 | 0.809 |
| risk | 0.946 | 0.938 | 0.894 | 0.949 | 0.939 | 0.933 |
| focuspast | 0.684 | 0.667 | 0.710 | 0.777 | 0.597 | 0.687 |
| focuspresent | 0.822 | 0.791 | 0.783 | 0.836 | 0.755 | 0.797 |
| focusfuture | 0.772 | 0.788 | 0.832 | 0.866 | 0.766 | 0.805 |
| relativ | 0.888 | 0.929 | 0.930 | 0.936 | 0.909 | 0.918 |
| motion | 0.770 | 0.755 | 0.731 | 0.805 | 0.796 | 0.771 |
| space | 0.915 | 0.914 | 0.908 | 0.935 | 0.884 | 0.911 |
| time | 0.950 | 0.956 | 0.968 | 0.963 | 0.954 | 0.958 |
| work | 0.946 | 0.946 | 0.942 | 0.959 | 0.955 | 0.950 |
| leisure | 0.909 | 0.889 | 0.846 | 0.911 | 0.859 | 0.883 |
| home | 0.765 | 0.755 | 0.773 | 0.796 | 0.778 | 0.774 |
| money | 0.927 | 0.896 | 0.967 | 0.823 | 0.925 | 0.908 |
| relig | 0.952 | 0.953 | 0.975 | 0.975 | 0.957 | 0.962 |
| death | 0.800 | 0.885 | 0.883 | 0.881 | 0.854 | 0.861 |
| informal | 0.929 | 0.867 | 0.877 | 0.924 | 0.856 | 0.891 |
| swear | 1.000 | 0.913 | 1.000 | 1.000 | 1.000 | 0.983 |
| netspeak | 0.982 | 0.980 | 0.982 | 0.971 | 0.970 | 0.977 |
| assent | 0.815 | 0.965 | 0.916 | 0.988 | 0.916 | 0.920 |
| nonflu | 0.759 | 0.378 | 0.371 | 0.752 | 0.357 | 0.523 |
| filler | . | . | . | . | . | . |
| allpunc | 0.913 | 0.928 | 0.948 | 0.933 | 0.889 | 0.922 |
| period | 0.933 | 0.747 | 0.971 | 0.661 | 0.430 | 0.748 |
| comma | 0.886 | 0.910 | 0.932 | 0.906 | 0.872 | 0.901 |
| colon | 0.905 | 0.905 | 0.959 | 0.597 | 0.869 | 0.847 |
| semic | 0.495 | 0.454 | 0.554 | 0.243 | 0.261 | 0.401 |
| qmark | . | . | . | . | . | . |
| exclam | . | . | . | . | . | . |
| dash | 0.756 | 0.805 | 0.758 | 0.810 | 0.871 | 0.800 |
| quote | . | . | . | 0.004 | . | 0.004 |
| apostro | . | . | . | . | . | . |
| parenth | 0.842 | 0.923 | 0.935 | 0.976 | 0.966 | 0.929 |
| otherp | 0.881 | 0.910 | 0.902 | 0.894 | 0.762 | 0.870 |
|  | 𝜌>.5 | 𝜌>.8 | 𝜌>.9 |  |  |  |
