# Supplementary material for: Automated content analysis across six languages
Source: PLoS One. 2019 Nov 20;14(11):e0224425. doi: 10.1371/journal.pone.0224425 (PMC6867602; doi:10.1371/journal.pone.0224425)
Supplement: S5 Table — (DOCX) [file pone.0224425.s005.docx]

S5 Table: Confidence intervals for the paired difference of LIWC variables (proportions) across languages

| LIWC Variable | English Mean | Language Translated From | | | | | | | | | |
| --- | --- | --- | --- | --- | --- | --- | --- | --- | --- | --- | --- |
|  |  | Arabic | | German | | French | | Russian | | Mandarin | |
| wc | 45.719 | 3.026 | 3.464 | 0.657 | 1.020 | 0.046 | 0.355 | -0.126 | 0.358 | -1.134 | -0.787 |
| analytic | 97.239 | -0.277 | 0.029 | -0.335 | -0.034 | -0.910 | -0.551 | 0.045 | 0.253 | -1.268 | -0.832 |
| clout | 64.062 | -0.338 | 0.108 | -0.290 | 0.215 | -1.260 | -0.740 | 0.023 | 0.452 | -1.501 | -0.857 |
| authentic | 32.315 | 3.124 | 4.305 | -2.198 | -1.158 | -2.910 | -1.814 | 0.154 | 1.061 | -1.680 | -0.434 |
| tone | 56.202 | -1.451 | -0.461 | -0.689 | 0.452 | -2.208 | -0.930 | -0.552 | 0.432 | -0.440 | 0.681 |
| wps | 45.011 | 2.785 | 3.300 | -9.718 | -8.664 | -0.416 | -0.014 | 0.274 | 0.831 | -13.782 | -12.533 |
| sixltr | 34.647 | -1.203 | -0.909 | -1.742 | -1.449 | -1.712 | -1.414 | -0.753 | -0.508 | -0.001 | 0.324 |
| dic | 73.599 | -0.313 | -0.003 | -0.551 | -0.265 | -0.325 | -0.043 | -0.547 | -0.301 | -3.619 | -3.279 |
| function | 40.456 | -0.534 | -0.250 | 0.251 | 0.520 | 0.606 | 0.882 | -0.190 | 0.050 | -4.835 | -4.501 |
| pronoun | 3.611 | -0.113 | 0.021 | 0.333 | 0.467 | 0.990 | 1.174 | 0.259 | 0.376 | -0.890 | -0.723 |
| ppron | 0.591 | -0.051 | 0.017 | 0.075 | 0.148 | 0.083 | 0.156 | 0.047 | 0.103 | -0.147 | -0.081 |
| i | 0.111 | -0.023 | -0.003 | -0.008 | 0.009 | -0.010 | 0.003 | -0.001 | 0.015 | -0.047 | -0.023 |
| we | 0.015 | 0.004 | 0.016 | -0.003 | 0.010 | 0.008 | 0.022 | 0.010 | 0.028 | 0.024 | 0.044 |
| you | 0.001 | 0.002 | 0.012 | 0.000 | 0.005 | 0.000 | 0.000 | -0.001 | 0.002 | 0.000 | 0.005 |
| shehe | 0.074 | 0.007 | 0.040 | 0.018 | 0.058 | 0.033 | 0.077 | 0.007 | 0.042 | -0.014 | 0.018 |
| they | 0.389 | -0.073 | -0.016 | 0.038 | 0.096 | 0.025 | 0.082 | 0.003 | 0.046 | -0.142 | -0.092 |
| ipron | 3.020 | -0.093 | 0.034 | 0.224 | 0.353 | 0.876 | 1.049 | 0.186 | 0.299 | -0.772 | -0.614 |
| article | 10.622 | -0.004 | 0.180 | 0.248 | 0.427 | -0.252 | -0.065 | -0.025 | 0.127 | -0.804 | -0.582 |
| prep | 19.021 | -0.682 | -0.489 | -0.760 | -0.552 | -0.731 | -0.509 | -0.225 | -0.042 | -3.650 | -3.360 |
| auxverb | 1.357 | -0.286 | -0.166 | 0.344 | 0.472 | 0.361 | 0.487 | -0.201 | -0.107 | -0.094 | 0.038 |
| adverb | 0.918 | -0.062 | 0.013 | -0.067 | 0.028 | -0.038 | 0.050 | -0.037 | 0.031 | -0.019 | 0.076 |
| conj | 5.755 | 0.224 | 0.382 | -0.329 | -0.220 | -0.287 | -0.179 | -0.184 | -0.101 | -0.348 | -0.219 |
| negate | 0.231 | -0.010 | 0.014 | -0.048 | -0.016 | 0.012 | 0.039 | -0.072 | -0.032 | 0.395 | 0.505 |
| verb | 3.704 | -0.146 | 0.009 | 0.336 | 0.509 | 0.457 | 0.640 | -0.260 | -0.126 | -0.106 | 0.077 |
| adj | 3.897 | -0.398 | -0.290 | -0.341 | -0.211 | -0.609 | -0.465 | -0.063 | 0.042 | -0.177 | -0.028 |
| compare | 1.361 | -0.242 | -0.161 | -0.230 | -0.126 | -0.271 | -0.164 | -0.036 | 0.050 | -0.202 | -0.084 |
| interrog | 0.373 | 0.011 | 0.066 | 0.056 | 0.118 | 0.035 | 0.091 | 0.014 | 0.063 | -0.177 | -0.115 |
| number | 8.840 | 0.547 | 0.739 | 0.767 | 0.898 | 0.194 | 0.288 | -0.018 | 0.080 | 1.632 | 1.794 |
| quant | 1.062 | 0.012 | 0.069 | 0.029 | 0.089 | 0.076 | 0.138 | 0.043 | 0.104 | 0.037 | 0.117 |
| affect | 4.190 | -0.215 | -0.112 | -0.121 | -0.005 | -0.143 | -0.019 | -0.066 | 0.033 | 0.122 | 0.235 |
| posemo | 3.153 | -0.193 | -0.111 | -0.114 | -0.013 | -0.196 | -0.087 | -0.056 | 0.026 | 0.058 | 0.151 |
| negemo | 0.978 | -0.044 | 0.015 | -0.026 | 0.029 | 0.022 | 0.083 | -0.022 | 0.033 | 0.044 | 0.106 |
| anx | 0.269 | -0.010 | 0.027 | -0.003 | 0.030 | 0.016 | 0.054 | -0.014 | 0.015 | 0.010 | 0.045 |
| anger | 0.392 | -0.023 | 0.009 | -0.032 | 0.003 | -0.012 | 0.026 | -0.022 | 0.009 | -0.010 | 0.026 |
| sad | 0.075 | -0.015 | 0.010 | -0.023 | -0.002 | -0.015 | 0.007 | -0.015 | 0.005 | -0.014 | 0.007 |
| social | 5.167 | -0.203 | -0.083 | -0.133 | 0.011 | -0.231 | -0.093 | -0.039 | 0.077 | 0.059 | 0.196 |
| family | 0.020 | -0.002 | 0.001 | 0.005 | 0.016 | -0.001 | 0.001 | -0.004 | 0.001 | 0.001 | 0.004 |
| friend | 0.122 | -0.039 | -0.018 | -0.043 | -0.016 | -0.005 | 0.011 | -0.029 | -0.006 | -0.030 | -0.003 |
| female | 0.095 | -0.014 | 0.003 | -0.004 | 0.019 | -0.010 | 0.010 | -0.008 | 0.010 | -0.006 | 0.007 |
| male | 0.118 | -0.011 | 0.023 | -0.013 | 0.028 | 0.086 | 0.133 | -0.004 | 0.032 | -0.037 | -0.002 |
| cogproc | 6.918 | -0.219 | -0.078 | -0.108 | 0.052 | 0.161 | 0.322 | -0.013 | 0.115 | 0.491 | 0.671 |
| insight | 3.318 | -0.199 | -0.121 | -0.109 | -0.016 | -0.061 | 0.032 | -0.071 | 0.006 | 0.228 | 0.350 |
| cause | 1.326 | -0.058 | 0.013 | -0.073 | 0.011 | -0.057 | 0.025 | -0.029 | 0.042 | 0.061 | 0.145 |
| discrep | 0.383 | -0.028 | 0.015 | -0.013 | 0.035 | 0.029 | 0.083 | 0.021 | 0.060 | 0.068 | 0.127 |
| tentat | 0.712 | 0.020 | 0.078 | -0.030 | 0.030 | -0.017 | 0.043 | -0.043 | 0.002 | -0.021 | 0.038 |
| certain | 0.956 | -0.029 | 0.031 | 0.063 | 0.130 | 0.138 | 0.213 | 0.009 | 0.058 | 0.000 | 0.068 |
| differ | 0.795 | -0.069 | -0.017 | -0.018 | 0.035 | 0.088 | 0.152 | -0.018 | 0.028 | 0.022 | 0.079 |
| percept | 0.303 | -0.035 | 0.001 | -0.030 | 0.008 | -0.044 | 0.004 | -0.102 | -0.056 | -0.030 | 0.012 |
| see | 0.222 | -0.036 | -0.008 | -0.041 | -0.011 | -0.069 | -0.035 | -0.107 | -0.065 | -0.037 | -0.005 |
| hear | 0.029 | -0.007 | 0.005 | 0.007 | 0.023 | 0.003 | 0.023 | -0.002 | 0.009 | 0.000 | 0.018 |
| feel | 0.043 | -0.003 | 0.013 | -0.009 | 0.005 | 0.011 | 0.034 | -0.005 | 0.007 | -0.003 | 0.011 |
| bio | 0.648 | -0.034 | 0.017 | -0.049 | -0.001 | -0.036 | 0.012 | -0.024 | 0.011 | -0.058 | -0.012 |
| body | 0.152 | -0.007 | 0.019 | -0.029 | -0.006 | -0.016 | 0.010 | -0.012 | 0.004 | -0.014 | 0.008 |
| health | 0.334 | -0.030 | 0.010 | -0.031 | 0.006 | -0.005 | 0.028 | -0.025 | 0.002 | -0.030 | 0.001 |
| sexual | 0.065 | -0.020 | -0.002 | -0.005 | 0.006 | -0.005 | 0.004 | -0.004 | 0.004 | 0.002 | 0.011 |
| ingest | 0.143 | -0.013 | 0.004 | -0.004 | 0.018 | -0.036 | -0.011 | -0.001 | 0.014 | -0.040 | -0.014 |
| drives | 7.628 | -0.077 | 0.062 | -0.054 | 0.098 | -0.208 | -0.046 | -0.128 | 0.007 | 0.346 | 0.505 |
| affiliation | 1.514 | -0.037 | 0.017 | -0.072 | -0.007 | 0.023 | 0.085 | -0.068 | -0.009 | 0.096 | 0.159 |
| achieve | 1.646 | 0.037 | 0.118 | -0.030 | 0.053 | -0.102 | -0.014 | -0.015 | 0.058 | 0.158 | 0.247 |
| power | 3.100 | -0.079 | 0.019 | -0.008 | 0.100 | 0.044 | 0.149 | -0.112 | -0.027 | 0.027 | 0.127 |
| reward | 0.797 | 0.074 | 0.136 | 0.066 | 0.133 | -0.006 | 0.070 | 0.034 | 0.095 | 0.018 | 0.092 |
| risk | 1.140 | -0.086 | -0.042 | -0.057 | -0.009 | -0.215 | -0.153 | -0.019 | 0.023 | 0.039 | 0.091 |
| focuspast | 0.808 | -0.009 | 0.075 | 0.052 | 0.141 | 0.102 | 0.188 | 0.001 | 0.072 | 0.103 | 0.205 |
| focuspresent | 2.608 | -0.094 | 0.021 | 0.257 | 0.385 | 0.330 | 0.465 | -0.236 | -0.128 | -0.097 | 0.050 |
| focusfuture | 0.525 | -0.066 | -0.008 | -0.057 | -0.003 | -0.068 | -0.015 | -0.077 | -0.036 | -0.040 | 0.019 |
| relativ | 13.256 | 0.915 | 1.152 | -0.365 | -0.160 | -0.666 | -0.453 | 0.133 | 0.316 | -0.654 | -0.421 |
| motion | 0.694 | 0.003 | 0.065 | 0.011 | 0.082 | -0.059 | 0.013 | -0.070 | -0.009 | -0.025 | 0.036 |
| space | 8.924 | -0.202 | -0.037 | -0.385 | -0.206 | -0.771 | -0.587 | 0.103 | 0.257 | -0.969 | -0.762 |
| time | 3.598 | 0.980 | 1.142 | -0.050 | 0.047 | 0.113 | 0.207 | 0.001 | 0.083 | 0.253 | 0.356 |
| work | 6.980 | -0.287 | -0.165 | -0.250 | -0.114 | -0.249 | -0.113 | -0.171 | -0.061 | 0.304 | 0.442 |
| leisure | 0.360 | -0.015 | 0.014 | -0.006 | 0.030 | -0.028 | 0.011 | -0.012 | 0.016 | -0.032 | 0.005 |
| home | 0.074 | -0.009 | 0.011 | -0.027 | -0.007 | -0.013 | 0.009 | -0.021 | -0.004 | -0.007 | 0.012 |
| money | 1.074 | -0.098 | -0.047 | -0.129 | -0.070 | -0.022 | 0.025 | -0.080 | -0.024 | -0.022 | 0.030 |
| relig | 0.109 | -0.013 | 0.001 | -0.018 | -0.002 | -0.007 | 0.005 | -0.016 | -0.002 | -0.005 | 0.011 |
| death | 0.078 | -0.029 | -0.005 | -0.019 | 0.000 | -0.020 | -0.002 | -0.014 | 0.002 | -0.010 | 0.009 |
| informal | 0.270 | -0.047 | -0.017 | -0.052 | -0.014 | -0.064 | -0.027 | 0.015 | 0.046 | -0.032 | 0.010 |
| swear | 0.002 | 0.000 | 0.000 | -0.001 | 0.002 | 0.000 | 0.000 | 0.000 | 0.000 | 0.000 | 0.000 |
| netspeak | 0.135 | -0.009 | 0.005 | 0.004 | 0.018 | 0.005 | 0.018 | 0.001 | 0.018 | 0.012 | 0.031 |
| assent | 0.008 | 0.000 | 0.010 | -0.001 | 0.003 | -0.002 | 0.004 | -0.001 | 0.000 | -0.003 | 0.004 |
| nonflu | 0.123 | -0.046 | -0.022 | -0.062 | -0.027 | -0.075 | -0.041 | 0.007 | 0.034 | -0.051 | -0.014 |
| filler |  |  |  |  |  |  |  |  |  |  |  |
| allpunc | 14.635 | 2.968 | 3.319 | -0.599 | -0.298 | -0.234 | 0.062 | -2.420 | -2.133 | 5.862 | 6.331 |
| period | 0.940 | -0.143 | -0.092 | 0.806 | 0.909 | -0.019 | 0.015 | -0.879 | -0.752 | 1.214 | 1.358 |
| comma | 6.343 | -0.398 | -0.197 | -1.140 | -0.947 | -0.636 | -0.460 | -1.112 | -0.925 | 0.831 | 1.079 |
| colon | 0.129 | -0.007 | 0.013 | -0.010 | 0.011 | 0.002 | 0.013 | -0.094 | -0.063 | 0.028 | 0.052 |
| semic | 0.925 | 0.792 | 0.929 | 0.451 | 0.567 | 0.799 | 0.928 | -0.162 | 0.010 | 2.091 | 2.319 |
| qmark |  |  |  |  |  |  |  |  |  |  |  |
| exclam |  |  |  |  |  |  |  |  |  |  |  |
| dash | 1.478 | 0.662 | 0.775 | -0.401 | -0.316 | -0.446 | -0.348 | -0.126 | -0.041 | -0.119 | -0.046 |
| quote | 0.802 | -0.869 | -0.735 | -0.869 | -0.735 | -0.869 | -0.735 | -0.865 | -0.731 | -0.869 | -0.735 |
| apostro | 0.175 | -0.197 | -0.152 | -0.197 | -0.152 | -0.197 | -0.152 | -0.197 | -0.152 | -0.197 | -0.152 |
| parenth | 2.326 | 0.568 | 0.754 | -0.193 | -0.090 | -0.228 | -0.113 | -0.307 | -0.226 | 0.082 | 0.154 |
| otherp | 1.517 | 2.187 | 2.398 | 0.638 | 0.772 | 1.057 | 1.219 | 0.956 | 1.113 | 2.426 | 2.676 |
