# Supplementary material for: Automated content analysis across six languages
Source: PLoS One. 2019 Nov 20;14(11):e0224425. doi: 10.1371/journal.pone.0224425 (PMC6867602; doi:10.1371/journal.pone.0224425)
Supplement: S6 Table — (DOCX) [file pone.0224425.s006.docx]

S6 Table: p-Values for t-test of paired differences from English

| LIWC Variable | Language Translated From | | | | |
| --- | --- | --- | --- | --- | --- |
|  | Arabic | German | French | Russian | Mandarin |
| wc | 0.000 | 0.000 | 0.011 | 0.347 | 0.000 |
| analytic | 0.113 | 0.017 | 0.000 | 0.005 | 0.000 |
| clout | 0.313 | 0.771 | 0.000 | 0.030 | 0.000 |
| authentic | 0.000 | 0.000 | 0.000 | 0.009 | 0.001 |
| tone | 0.000 | 0.684 | 0.000 | 0.811 | 0.674 |
| wps | 0.000 | 0.000 | 0.036 | 0.000 | 0.000 |
| sixltr | 0.000 | 0.000 | 0.000 | 0.000 | 0.051 |
| dic | 0.045 | 0.000 | 0.010 | 0.000 | 0.000 |
| function | 0.000 | 0.000 | 0.000 | 0.252 | 0.000 |
| pronoun | 0.176 | 0.000 | 0.000 | 0.000 | 0.000 |
| ppron | 0.326 | 0.000 | 0.000 | 0.000 | 0.000 |
| i | 0.012 | 0.926 | 0.265 | 0.074 | 0.000 |
| we | 0.002 | 0.243 | 0.000 | 0.000 | 0.000 |
| you | 0.003 | 0.057 | 0.157 | 0.326 | 0.064 |
| shehe | 0.005 | 0.000 | 0.000 | 0.006 | 0.820 |
| they | 0.002 | 0.000 | 0.000 | 0.028 | 0.000 |
| ipron | 0.368 | 0.000 | 0.000 | 0.000 | 0.000 |
| article | 0.060 | 0.000 | 0.001 | 0.190 | 0.000 |
| prep | 0.000 | 0.000 | 0.000 | 0.004 | 0.000 |
| auxverb | 0.000 | 0.000 | 0.000 | 0.000 | 0.398 |
| adverb | 0.205 | 0.425 | 0.797 | 0.863 | 0.236 |
| conj | 0.000 | 0.000 | 0.000 | 0.000 | 0.000 |
| negate | 0.759 | 0.000 | 0.000 | 0.000 | 0.000 |
| verb | 0.083 | 0.000 | 0.000 | 0.000 | 0.755 |
| adj | 0.000 | 0.000 | 0.000 | 0.696 | 0.007 |
| compare | 0.000 | 0.000 | 0.000 | 0.762 | 0.000 |
| interrog | 0.007 | 0.000 | 0.000 | 0.002 | 0.000 |
| number | 0.000 | 0.000 | 0.000 | 0.213 | 0.000 |
| quant | 0.006 | 0.000 | 0.000 | 0.000 | 0.000 |
| affect | 0.000 | 0.034 | 0.010 | 0.516 | 0.000 |
| posemo | 0.000 | 0.014 | 0.000 | 0.475 | 0.000 |
| negemo | 0.341 | 0.923 | 0.001 | 0.691 | 0.000 |
| anx | 0.383 | 0.102 | 0.000 | 0.915 | 0.002 |
| anger | 0.405 | 0.100 | 0.456 | 0.447 | 0.367 |
| sad | 0.661 | 0.022 | 0.501 | 0.351 | 0.531 |
| social | 0.000 | 0.097 | 0.000 | 0.516 | 0.000 |
| family | 0.494 | 0.000 | 0.734 | 0.167 | 0.005 |
| friend | 0.000 | 0.000 | 0.478 | 0.002 | 0.014 |
| female | 0.178 | 0.219 | 0.973 | 0.815 | 0.917 |
| male | 0.518 | 0.467 | 0.000 | 0.120 | 0.028 |
| cogproc | 0.000 | 0.498 | 0.000 | 0.119 | 0.000 |
| insight | 0.000 | 0.009 | 0.536 | 0.097 | 0.000 |
| cause | 0.216 | 0.150 | 0.456 | 0.730 | 0.000 |
| discrep | 0.554 | 0.362 | 0.000 | 0.000 | 0.000 |
| tentat | 0.001 | 0.984 | 0.405 | 0.077 | 0.581 |
| certain | 0.939 | 0.000 | 0.000 | 0.007 | 0.053 |
| differ | 0.001 | 0.530 | 0.000 | 0.652 | 0.001 |
| percept | 0.060 | 0.248 | 0.098 | 0.000 | 0.409 |
| see | 0.003 | 0.001 | 0.000 | 0.000 | 0.009 |
| hear | 0.652 | 0.000 | 0.013 | 0.171 | 0.055 |
| feel | 0.221 | 0.524 | 0.000 | 0.758 | 0.304 |
| bio | 0.528 | 0.041 | 0.340 | 0.481 | 0.003 |
| body | 0.378 | 0.003 | 0.617 | 0.312 | 0.615 |
| health | 0.320 | 0.184 | 0.183 | 0.083 | 0.074 |
| sexual | 0.017 | 0.873 | 0.736 | 0.954 | 0.004 |
| ingest | 0.298 | 0.227 | 0.000 | 0.103 | 0.000 |
| drives | 0.828 | 0.575 | 0.002 | 0.077 | 0.000 |
| affiliation | 0.465 | 0.017 | 0.001 | 0.011 | 0.000 |
| achieve | 0.000 | 0.585 | 0.009 | 0.245 | 0.000 |
| power | 0.229 | 0.097 | 0.000 | 0.001 | 0.003 |
| reward | 0.000 | 0.000 | 0.101 | 0.000 | 0.003 |
| risk | 0.000 | 0.008 | 0.000 | 0.838 | 0.000 |
| focuspast | 0.128 | 0.000 | 0.000 | 0.042 | 0.000 |
| focuspresent | 0.216 | 0.000 | 0.000 | 0.000 | 0.534 |
| focusfuture | 0.013 | 0.030 | 0.002 | 0.000 | 0.497 |
| relativ | 0.000 | 0.000 | 0.000 | 0.000 | 0.000 |
| motion | 0.034 | 0.010 | 0.207 | 0.011 | 0.710 |
| space | 0.005 | 0.000 | 0.000 | 0.000 | 0.000 |
| time | 0.000 | 0.955 | 0.000 | 0.046 | 0.000 |
| work | 0.000 | 0.000 | 0.000 | 0.000 | 0.000 |
| leisure | 0.984 | 0.183 | 0.382 | 0.794 | 0.162 |
| home | 0.858 | 0.001 | 0.688 | 0.006 | 0.606 |
| money | 0.000 | 0.000 | 0.910 | 0.000 | 0.768 |
| relig | 0.115 | 0.010 | 0.737 | 0.014 | 0.498 |
| death | 0.005 | 0.040 | 0.019 | 0.141 | 0.921 |
| informal | 0.000 | 0.001 | 0.000 | 0.000 | 0.320 |
| swear | 0.048 | 0.402 | 0.655 | 0.180 | 0.185 |
| netspeak | 0.582 | 0.001 | 0.001 | 0.021 | 0.000 |
| assent | 0.032 | 0.177 | 0.580 | 0.218 | 0.801 |
| nonflu | 0.000 | 0.000 | 0.000 | 0.002 | 0.000 |
| filler |  |  |  |  |  |
| allpunc | 0.000 | 0.000 | 0.255 | 0.000 | 0.000 |
| period | 0.000 | 0.000 | 0.821 | 0.000 | 0.000 |
| comma | 0.000 | 0.000 | 0.000 | 0.000 | 0.000 |
| colon | 0.570 | 0.976 | 0.011 | 0.000 | 0.000 |
| semic | 0.000 | 0.000 | 0.000 | 0.084 | 0.000 |
| qmark |  |  |  |  |  |
| exclam |  |  |  |  |  |
| dash | 0.000 | 0.000 | 0.000 | 0.000 | 0.000 |
| quote | 0.000 | 0.000 | 0.000 | 0.000 | 0.000 |
| apostro | 0.000 | 0.000 | 0.000 | 0.000 | 0.000 |
| parenth | 0.000 | 0.000 | 0.000 | 0.000 | 0.000 |
| otherp | 0.000 | 0.000 | 0.000 | 0.000 | 0.000 |
|  | p>0.05 |  |  |  |  |
