# Supplementary material for: Automated content analysis across six languages
Source: PLoS One. 2019 Nov 20;14(11):e0224425. doi: 10.1371/journal.pone.0224425 (PMC6867602; doi:10.1371/journal.pone.0224425)
Supplement: S7 Table — (DOCX) [file pone.0224425.s007.docx]

S7 Table: Confidence intervals for the paired difference of LIWC variables (proportions) across languages expressed as percentages of the English mean.

| LIWC Variable | English Mean | Language Translated From | | | | | | | | | |
| --- | --- | --- | --- | --- | --- | --- | --- | --- | --- | --- | --- |
|  |  | Arabic | | German | | French | | Russian | | Mandarin | |
| wc | 45.719 | 6.6% | 7.6% | 1.4% | 2.2% | 0.1% | 0.8% | -0.3% | 0.8% | -2.5% | -1.7% |
| analytic | 97.239 | -0.3% | 0.0% | -0.3% | 0.0% | -0.9% | -0.6% | 0.0% | 0.3% | -1.3% | -0.9% |
| clout | 64.062 | -0.5% | 0.2% | -0.5% | 0.3% | -2.0% | -1.2% | 0.0% | 0.7% | -2.3% | -1.3% |
| authentic | 32.315 | 9.7% | 13.3% | -6.8% | -3.6% | -9.0% | -5.6% | 0.5% | 3.3% | -5.2% | -1.3% |
| tone | 56.202 | -2.6% | -0.8% | -1.2% | 0.8% | -3.9% | -1.7% | -1.0% | 0.8% | -0.8% | 1.2% |
| wps | 45.011 | 6.2% | 7.3% | -21.6% | -19.2% | -0.9% | 0.0% | 0.6% | 1.8% | -30.6% | -27.8% |
| sixltr | 34.647 | -3.5% | -2.6% | -5.0% | -4.2% | -4.9% | -4.1% | -2.2% | -1.5% | 0.0% | 0.9% |
| dic | 73.599 | -0.4% | 0.0% | -0.7% | -0.4% | -0.4% | -0.1% | -0.7% | -0.4% | -4.9% | -4.5% |
| function | 40.456 | -1.3% | -0.6% | 0.6% | 1.3% | 1.5% | 2.2% | -0.5% | 0.1% | -12.0% | -11.1% |
| pronoun | 3.611 | -3.1% | 0.6% | 9.2% | 12.9% | 27.4% | 32.5% | 7.2% | 10.4% | -24.7% | -20.0% |
| ppron | 0.591 | -8.6% | 2.9% | 12.8% | 25.0% | 14.1% | 26.4% | 8.0% | 17.4% | -24.8% | -13.8% |
| i | 0.111 | -21.1% | -2.6% | -7.6% | 8.4% | -9.4% | 2.6% | -0.6% | 13.4% | -42.4% | -20.8% |
| we | 0.015 | 24.6% | 108.1% | -17.4% | 68.6% | 53.6% | 148.5% | 63.5% | 184.4% | 159.7% | 294.5% |
| you | 0.001 | 392.3% | 1992.2% | -12.8% | 887.2% | -4.6% | 0.7% | -107.3% | 322.8% | -20.8% | 760.0% |
| shehe | 0.074 | 9.4% | 53.9% | 23.9% | 77.4% | 44.7% | 103.6% | 9.4% | 55.9% | -19.2% | 24.2% |
| they | 0.389 | -18.7% | -4.1% | 9.7% | 24.7% | 6.4% | 21.0% | 0.7% | 11.8% | -36.5% | -23.5% |
| ipron | 3.020 | -3.1% | 1.1% | 7.4% | 11.7% | 29.0% | 34.7% | 6.2% | 9.9% | -25.6% | -20.3% |
| article | 10.622 | 0.0% | 1.7% | 2.3% | 4.0% | -2.4% | -0.6% | -0.2% | 1.2% | -7.6% | -5.5% |
| prep | 19.021 | -3.6% | -2.6% | -4.0% | -2.9% | -3.8% | -2.7% | -1.2% | -0.2% | -19.2% | -17.7% |
| auxverb | 1.357 | -21.1% | -12.2% | 25.3% | 34.8% | 26.6% | 35.9% | -14.8% | -7.9% | -7.0% | 2.8% |
| adverb | 0.918 | -6.8% | 1.5% | -7.3% | 3.1% | -4.2% | 5.5% | -4.0% | 3.4% | -2.0% | 8.3% |
| conj | 5.755 | 3.9% | 6.6% | -5.7% | -3.8% | -5.0% | -3.1% | -3.2% | -1.8% | -6.0% | -3.8% |
| negate | 0.231 | -4.5% | 6.2% | -21.0% | -7.1% | 5.4% | 17.1% | -31.4% | -13.8% | 171.1% | 219.1% |
| verb | 3.704 | -3.9% | 0.2% | 9.1% | 13.7% | 12.3% | 17.3% | -7.0% | -3.4% | -2.9% | 2.1% |
| adj | 3.897 | -10.2% | -7.4% | -8.8% | -5.4% | -15.6% | -11.9% | -1.6% | 1.1% | -4.5% | -0.7% |
| compare | 1.361 | -17.8% | -11.8% | -16.9% | -9.2% | -19.9% | -12.0% | -2.7% | 3.6% | -14.9% | -6.2% |
| interrog | 0.373 | 2.8% | 17.8% | 14.9% | 31.7% | 9.3% | 24.4% | 3.8% | 16.9% | -47.5% | -30.7% |
| number | 8.840 | 6.2% | 8.4% | 8.7% | 10.2% | 2.2% | 3.3% | -0.2% | 0.9% | 18.5% | 20.3% |
| quant | 1.062 | 1.1% | 6.5% | 2.7% | 8.4% | 7.2% | 13.0% | 4.1% | 9.8% | 3.5% | 11.0% |
| affect | 4.190 | -5.1% | -2.7% | -2.9% | -0.1% | -3.4% | -0.5% | -1.6% | 0.8% | 2.9% | 5.6% |
| posemo | 3.153 | -6.1% | -3.5% | -3.6% | -0.4% | -6.2% | -2.8% | -1.8% | 0.8% | 1.8% | 4.8% |
| negemo | 0.978 | -4.5% | 1.6% | -2.7% | 3.0% | 2.2% | 8.5% | -2.2% | 3.3% | 4.5% | 10.8% |
| anx | 0.269 | -3.9% | 10.0% | -1.0% | 11.0% | 5.8% | 20.0% | -5.1% | 5.7% | 3.8% | 16.9% |
| anger | 0.392 | -5.8% | 2.3% | -8.1% | 0.7% | -3.0% | 6.7% | -5.5% | 2.4% | -2.5% | 6.6% |
| sad | 0.075 | -20.2% | 12.8% | -30.6% | -2.3% | -20.5% | 10.0% | -20.0% | 7.1% | -18.9% | 9.7% |
| social | 5.167 | -3.9% | -1.6% | -2.6% | 0.2% | -4.5% | -1.8% | -0.8% | 1.5% | 1.1% | 3.8% |
| family | 0.020 | -8.7% | 4.2% | 23.1% | 78.5% | -3.4% | 4.9% | -22.0% | 3.8% | 3.9% | 22.1% |
| friend | 0.122 | -32.3% | -14.3% | -35.3% | -13.4% | -4.3% | 9.1% | -23.4% | -5.1% | -24.2% | -2.7% |
| female | 0.095 | -14.7% | 2.7% | -4.6% | 20.2% | -10.7% | 10.4% | -8.3% | 10.6% | -6.5% | 7.3% |
| male | 0.118 | -9.7% | 19.3% | -11.0% | 24.1% | 73.2% | 112.9% | -3.1% | 27.3% | -31.9% | -1.8% |
| cogproc | 6.918 | -3.2% | -1.1% | -1.6% | 0.8% | 2.3% | 4.7% | -0.2% | 1.7% | 7.1% | 9.7% |
| insight | 3.318 | -6.0% | -3.6% | -3.3% | -0.5% | -1.8% | 1.0% | -2.1% | 0.2% | 6.9% | 10.5% |
| cause | 1.326 | -4.4% | 1.0% | -5.5% | 0.8% | -4.3% | 1.9% | -2.2% | 3.2% | 4.6% | 10.9% |
| discrep | 0.383 | -7.4% | 4.0% | -3.3% | 9.0% | 7.5% | 21.8% | 5.5% | 15.6% | 17.7% | 33.2% |
| tentat | 0.712 | 2.8% | 11.0% | -4.2% | 4.3% | -2.5% | 6.1% | -6.1% | 0.3% | -3.0% | 5.3% |
| certain | 0.956 | -3.0% | 3.3% | 6.6% | 13.6% | 14.4% | 22.3% | 0.9% | 6.0% | -0.1% | 7.1% |
| differ | 0.795 | -8.6% | -2.1% | -2.3% | 4.4% | 11.1% | 19.2% | -2.2% | 3.5% | 2.8% | 10.0% |
| percept | 0.303 | -11.6% | 0.2% | -9.9% | 2.6% | -14.4% | 1.2% | -33.7% | -18.4% | -9.8% | 4.0% |
| see | 0.222 | -16.3% | -3.4% | -18.5% | -5.1% | -31.1% | -15.9% | -48.2% | -29.1% | -16.8% | -2.3% |
| hear | 0.029 | -25.2% | 15.8% | 23.3% | 81.5% | 9.8% | 81.6% | -5.9% | 33.3% | -0.7% | 64.1% |
| feel | 0.043 | -6.9% | 29.9% | -20.9% | 10.7% | 24.6% | 79.7% | -11.5% | 15.8% | -8.0% | 25.5% |
| bio | 0.648 | -5.2% | 2.7% | -7.6% | -0.2% | -5.5% | 1.9% | -3.7% | 1.7% | -9.0% | -1.8% |
| body | 0.152 | -4.8% | 12.7% | -18.8% | -3.9% | -10.6% | 6.3% | -8.0% | 2.6% | -9.2% | 5.5% |
| health | 0.334 | -8.8% | 2.9% | -9.3% | 1.8% | -1.6% | 8.3% | -7.6% | 0.5% | -8.9% | 0.4% |
| sexual | 0.065 | -31.4% | -3.1% | -8.1% | 9.5% | -7.9% | 5.6% | -6.8% | 6.4% | 3.4% | 17.5% |
| ingest | 0.143 | -9.1% | 2.8% | -3.0% | 12.5% | -24.8% | -7.4% | -0.9% | 9.9% | -27.8% | -9.9% |
| drives | 7.628 | -1.0% | 0.8% | -0.7% | 1.3% | -2.7% | -0.6% | -1.7% | 0.1% | 4.5% | 6.6% |
| affiliation | 1.514 | -2.4% | 1.1% | -4.7% | -0.5% | 1.5% | 5.6% | -4.5% | -0.6% | 6.3% | 10.5% |
| achieve | 1.646 | 2.3% | 7.2% | -1.8% | 3.2% | -6.2% | -0.9% | -0.9% | 3.5% | 9.6% | 15.0% |
| power | 3.100 | -2.6% | 0.6% | -0.3% | 3.2% | 1.4% | 4.8% | -3.6% | -0.9% | 0.9% | 4.1% |
| reward | 0.797 | 9.3% | 17.0% | 8.2% | 16.7% | -0.8% | 8.7% | 4.3% | 12.0% | 2.3% | 11.6% |
| risk | 1.140 | -7.6% | -3.7% | -5.0% | -0.8% | -18.9% | -13.5% | -1.7% | 2.0% | 3.4% | 8.0% |
| focuspast | 0.808 | -1.2% | 9.3% | 6.4% | 17.5% | 12.6% | 23.2% | 0.2% | 8.8% | 12.8% | 25.3% |
| focuspresent | 2.608 | -3.6% | 0.8% | 9.8% | 14.8% | 12.7% | 17.8% | -9.0% | -4.9% | -3.7% | 1.9% |
| focusfuture | 0.525 | -12.5% | -1.5% | -10.8% | -0.5% | -13.0% | -2.9% | -14.7% | -6.8% | -7.6% | 3.7% |
| relativ | 13.256 | 6.9% | 8.7% | -2.8% | -1.2% | -5.0% | -3.4% | 1.0% | 2.4% | -4.9% | -3.2% |
| motion | 0.694 | 0.4% | 9.4% | 1.6% | 11.8% | -8.5% | 1.8% | -10.1% | -1.3% | -3.6% | 5.2% |
| space | 8.924 | -2.3% | -0.4% | -4.3% | -2.3% | -8.6% | -6.6% | 1.2% | 2.9% | -10.9% | -8.5% |
| time | 3.598 | 27.2% | 31.7% | -1.4% | 1.3% | 3.1% | 5.8% | 0.0% | 2.3% | 7.0% | 9.9% |
| work | 6.980 | -4.1% | -2.4% | -3.6% | -1.6% | -3.6% | -1.6% | -2.4% | -0.9% | 4.4% | 6.3% |
| leisure | 0.360 | -4.1% | 4.0% | -1.6% | 8.3% | -7.8% | 3.0% | -3.3% | 4.4% | -8.9% | 1.5% |
| home | 0.074 | -11.9% | 14.3% | -37.1% | -9.1% | -17.7% | 11.7% | -28.6% | -4.8% | -9.8% | 16.8% |
| money | 1.074 | -9.1% | -4.4% | -12.0% | -6.6% | -2.1% | 2.3% | -7.4% | -2.2% | -2.0% | 2.8% |
| relig | 0.109 | -11.9% | 1.3% | -16.9% | -2.3% | -6.0% | 4.3% | -14.6% | -1.6% | -4.8% | 9.8% |
| death | 0.078 | -36.7% | -6.6% | -24.8% | -0.6% | -25.9% | -2.3% | -18.4% | 2.6% | -13.0% | 11.7% |
| informal | 0.270 | -17.3% | -6.4% | -19.2% | -5.2% | -23.8% | -9.9% | 5.5% | 17.1% | -11.7% | 3.8% |
| swear | 0.002 | -11.8% | -0.1% | -31.2% | 77.9% | -1.4% | 2.2% | -0.2% | 1.0% | -0.7% | 3.8% |
| netspeak | 0.135 | -6.6% | 3.7% | 3.3% | 13.3% | 3.5% | 13.2% | 1.1% | 13.3% | 8.9% | 23.2% |
| assent | 0.008 | 5.8% | 126.5% | -7.5% | 40.8% | -29.8% | 53.1% | -11.7% | 2.7% | -38.8% | 50.3% |
| nonflu | 0.123 | -37.1% | -17.6% | -50.1% | -21.9% | -60.9% | -33.1% | 6.0% | 27.6% | -41.2% | -11.6% |
| filler | 0 |  |  |  |  |  |  |  |  |  |  |
| allpunc | 14.635 | 20.3% | 22.7% | -4.1% | -2.0% | -1.6% | 0.4% | -16.5% | -14.6% | 40.1% | 43.3% |
| period | 0.940 | -15.2% | -9.8% | 85.8% | 96.8% | -2.0% | 1.6% | -93.5% | -80.0% | 129.2% | 144.5% |
| comma | 6.343 | -6.3% | -3.1% | -18.0% | -14.9% | -10.0% | -7.3% | -17.5% | -14.6% | 13.1% | 17.0% |
| colon | 0.129 | -5.5% | 10.0% | -8.1% | 8.3% | 1.4% | 10.5% | -73.4% | -49.1% | 22.1% | 40.2% |
| semic | 0.925 | 85.6% | 100.5% | 48.8% | 61.3% | 86.4% | 100.3% | -17.5% | 1.1% | 226.1% | 250.8% |
| qmark | 0 |  |  |  |  |  |  |  |  |  |  |
| exclam | 0 |  |  |  |  |  |  |  |  |  |  |
| dash | 1.478 | 44.8% | 52.4% | -27.1% | -21.4% | -30.2% | -23.6% | -8.5% | -2.8% | -8.1% | -3.1% |
| quote | 0.802 | -108.4% | -91.6% | -108.4% | -91.6% | -108.4% | -91.6% | -107.9% | -91.1% | -108.4% | -91.6% |
| apostro | 0.175 | -112.8% | -87.2% | -112.8% | -87.2% | -112.8% | -87.2% | -112.8% | -87.2% | -112.8% | -87.2% |
| parenth | 2.326 | 24.4% | 32.4% | -8.3% | -3.9% | -9.8% | -4.9% | -13.2% | -9.7% | 3.5% | 6.6% |
| otherp | 1.517 | 144.2% | 158.1% | 42.1% | 50.9% | 69.7% | 80.3% | 63.0% | 73.4% | 160.0% | 176.4% |
|  | p>0.05 |  |  |  |  |  |  |  |  |  |  |
