# Supplementary material for: Automated content analysis across six languages
Source: PLoS One. 2019 Nov 20;14(11):e0224425. doi: 10.1371/journal.pone.0224425 (PMC6867602; doi:10.1371/journal.pone.0224425)
Supplement: S8 Table — (DOCX) [file pone.0224425.s008.docx]

S8 Table. Cohen’s d effect sizes across languages

| LIWC Variable | Language Translated From | | | | |
| --- | --- | --- | --- | --- | --- |
|  | Arabic | German | French | Russian | Mandarin |
| wc | 0.148 | 0.038 | 0.009 | 0.005 | -0.044 |
| analytic | -0.021 | -0.031 | -0.124 | 0.025 | -0.179 |
| clout | -0.007 | -0.002 | -0.064 | 0.015 | -0.075 |
| authentic | 0.138 | -0.062 | -0.088 | 0.023 | -0.039 |
| tone | -0.028 | -0.003 | -0.045 | -0.002 | 0.003 |
| wps | 0.135 | -0.409 | -0.010 | 0.025 | -0.586 |
| sixltr | -0.121 | -0.183 | -0.179 | -0.072 | 0.019 |
| dic | -0.014 | -0.036 | -0.016 | -0.038 | -0.305 |
| function | -0.050 | 0.049 | 0.095 | -0.009 | -0.598 |
| pronoun | -0.015 | 0.127 | 0.345 | 0.101 | -0.257 |
| ppron | -0.012 | 0.080 | 0.086 | 0.054 | -0.082 |
| i | -0.018 | 0.001 | -0.005 | 0.010 | -0.048 |
| we | 0.040 | 0.015 | 0.061 | 0.075 | 0.137 |
| you | 0.287 | 0.105 | 0.000 | 0.026 | 0.089 |
| shehe | 0.047 | 0.075 | 0.109 | 0.048 | 0.004 |
| they | -0.041 | 0.061 | 0.049 | 0.022 | -0.107 |
| ipron | -0.010 | 0.099 | 0.330 | 0.083 | -0.238 |
| article | 0.018 | 0.067 | -0.032 | 0.010 | -0.138 |
| prep | -0.117 | -0.131 | -0.124 | -0.027 | -0.701 |
| auxverb | -0.097 | 0.175 | 0.182 | -0.066 | -0.012 |
| adverb | -0.015 | -0.012 | 0.004 | -0.002 | 0.018 |
| conj | 0.081 | -0.074 | -0.063 | -0.038 | -0.076 |
| negate | 0.002 | -0.039 | 0.031 | -0.063 | 0.541 |
| verb | -0.020 | 0.123 | 0.160 | -0.056 | -0.004 |
| adj | -0.102 | -0.082 | -0.159 | -0.003 | -0.030 |
| compare | -0.098 | -0.087 | -0.106 | 0.003 | -0.070 |
| interrog | 0.038 | 0.086 | 0.062 | 0.038 | -0.145 |
| number | 0.052 | 0.067 | 0.019 | 0.002 | 0.137 |
| quant | 0.022 | 0.033 | 0.059 | 0.041 | 0.043 |
| affect | -0.039 | -0.015 | -0.019 | -0.004 | 0.042 |
| posemo | -0.045 | -0.019 | -0.042 | -0.004 | 0.031 |
| negemo | -0.007 | 0.001 | 0.024 | 0.002 | 0.034 |
| anx | 0.009 | 0.014 | 0.036 | 0.001 | 0.029 |
| anger | -0.005 | -0.011 | 0.006 | -0.005 | 0.006 |
| sad | -0.005 | -0.024 | -0.007 | -0.009 | -0.007 |
| social | -0.033 | -0.014 | -0.038 | 0.004 | 0.030 |
| family | -0.002 | 0.039 | 0.001 | -0.007 | 0.010 |
| friend | -0.048 | -0.050 | 0.005 | -0.029 | -0.028 |
| female | -0.009 | 0.012 | 0.000 | 0.002 | 0.001 |
| male | 0.009 | 0.013 | 0.183 | 0.024 | -0.033 |
| cogproc | -0.032 | -0.006 | 0.051 | 0.011 | 0.124 |
| insight | -0.050 | -0.020 | -0.005 | -0.010 | 0.091 |
| cause | -0.011 | -0.016 | -0.008 | 0.003 | 0.051 |
| discrep | -0.006 | 0.011 | 0.054 | 0.039 | 0.094 |
| tentat | 0.029 | 0.000 | 0.008 | -0.012 | 0.005 |
| certain | 0.001 | 0.055 | 0.101 | 0.019 | 0.019 |
| differ | -0.024 | 0.005 | 0.068 | 0.003 | 0.029 |
| percept | -0.017 | -0.011 | -0.020 | -0.077 | -0.009 |
| see | -0.026 | -0.032 | -0.063 | -0.103 | -0.026 |
| hear | -0.005 | 0.051 | 0.045 | 0.013 | 0.031 |
| feel | 0.013 | -0.006 | 0.059 | 0.002 | 0.010 |
| bio | -0.005 | -0.016 | -0.007 | -0.004 | -0.022 |
| body | 0.009 | -0.027 | -0.005 | -0.006 | -0.004 |
| health | -0.008 | -0.010 | 0.009 | -0.009 | -0.011 |
| sexual | -0.019 | 0.001 | -0.001 | 0.000 | 0.011 |
| ingest | -0.007 | 0.010 | -0.034 | 0.010 | -0.040 |
| drives | -0.001 | 0.004 | -0.024 | -0.011 | 0.080 |
| affiliation | -0.005 | -0.018 | 0.025 | -0.018 | 0.059 |
| achieve | 0.032 | 0.005 | -0.024 | 0.009 | 0.084 |
| power | -0.009 | 0.014 | 0.030 | -0.022 | 0.024 |
| reward | 0.070 | 0.066 | 0.021 | 0.043 | 0.037 |
| risk | -0.031 | -0.016 | -0.089 | 0.001 | 0.031 |
| focuspast | 0.021 | 0.064 | 0.095 | 0.024 | 0.101 |
| focuspresent | -0.013 | 0.113 | 0.140 | -0.064 | -0.008 |
| focusfuture | -0.029 | -0.024 | -0.033 | -0.045 | -0.008 |
| relativ | 0.190 | -0.048 | -0.103 | 0.041 | -0.099 |
| motion | 0.024 | 0.034 | -0.017 | -0.028 | 0.004 |
| space | -0.024 | -0.059 | -0.135 | 0.036 | -0.173 |
| time | 0.276 | 0.000 | 0.042 | 0.011 | 0.079 |
| work | -0.044 | -0.036 | -0.035 | -0.023 | 0.073 |
| leisure | 0.000 | 0.012 | -0.008 | 0.002 | -0.013 |
| home | 0.002 | -0.036 | -0.005 | -0.026 | 0.005 |
| money | -0.034 | -0.047 | 0.001 | -0.025 | 0.002 |
| relig | -0.009 | -0.016 | -0.001 | -0.014 | 0.004 |
| death | -0.033 | -0.019 | -0.021 | -0.012 | -0.001 |
| informal | -0.037 | -0.038 | -0.052 | 0.035 | -0.012 |
| swear | -0.002 | 0.008 | 0.000 | 0.000 | 0.001 |
| netspeak | -0.003 | 0.016 | 0.016 | 0.014 | 0.031 |
| assent | 0.030 | 0.007 | 0.005 | -0.002 | 0.003 |
| nonflu | -0.065 | -0.086 | -0.112 | 0.040 | -0.063 |
| Overall | 0.134 | 0.020 | 0.027 | -0.121 | 0.166 |
| Effect Size | Very Small | Small | Medium | Large |  |
